# Adrenal hormones mediate disease tolerance in malaria

**DOI:** 10.1038/s41467-018-06986-5

**Published:** 2018-10-30

**Authors:** Leen Vandermosten, Thao-Thy Pham, Sofie Knoops, Charlotte De Geest, Natacha Lays, Kristof Van der Molen, Christopher J. Kenyon, Manu Verma, Karen E. Chapman, Frans Schuit, Karolien De Bosscher, Ghislain Opdenakker, Philippe E. Van den Steen

**Affiliations:** 1 Laboratory of Immunobiology, Department of Microbiology and Immunology, Rega Institute for Medical Research, KU Leuven, Leuven, 3000 Belgium; 2 Centre for Cardiovascular Science, The Queen’s Medical Research Institute, University of Edinburgh, Edinburgh, EH16 4TJ United Kingdom; 3 Gene Expression Unit, Department of Cellular and Molecular Medicine, KU Leuven, Leuven, 3000 Belgium; 4 Nuclear Receptor Lab, Receptor Research Laboratories, VIB Center for Medical Biotechnology, Ghent University, Gent, 9000 Belgium

## Abstract

Malaria reduces host fitness and survival by pathogen-mediated damage and inflammation. Disease tolerance mechanisms counter these negative effects without decreasing pathogen load. Here, we demonstrate that in four different mouse models of malaria, adrenal hormones confer disease tolerance and protect against early death, independently of parasitemia. Surprisingly, adrenalectomy differentially affects malaria-induced inflammation by increasing circulating cytokines and inflammation in the brain but not in the liver or lung. Furthermore, without affecting the transcription of hepatic gluconeogenic enzymes, adrenalectomy causes exhaustion of hepatic glycogen and insulin-independent lethal hypoglycemia upon infection. This hypoglycemia is not prevented by glucose administration or TNF-α neutralization. In contrast, treatment with a synthetic glucocorticoid (dexamethasone) prevents the hypoglycemia, lowers cerebral cytokine expression and increases survival rates. Overall, we conclude that in malaria, adrenal hormones do not protect against lung and liver inflammation. Instead, they prevent excessive systemic and brain inflammation and severe hypoglycemia, thereby contributing to tolerance.

## Abstract (web summary)

Disease tolerance mechanisms counter the negative effects of infection without decreasing the pathogen load. Here, the authors show that in mouse models of malaria, such disease tolerance can be conferred by adrenal hormones, by preventing excessive inflammation and hypoglycemia.

## Introduction

Malaria is a devastating parasitic disease, leading to an estimated 216 million clinical cases and 445,000 deaths in 2016^1^. The infection can evolve as an uncomplicated febrile disease or develop into complications that include cerebral malaria (CM), severe malarial anemia, placental malaria, hypoglycemia and malaria-associated acute respiratory distress syndrome (MA-ARDS). These complications cannot be efficiently cured by current antimalarial drugs, despite the effective inhibition of parasite growth. Complicated malaria has a mortality of about 15% for CM and up to 80% for MA-ARDS^2,3^. Malarial complications can be inflicted by the parasite and/or by an exaggerated immune reaction^4^. Therefore, protection against malaria complications not only involves pathogen clearance. Also host defense mechanisms that do not interfere with the pathogen load enable the host to limit the consequences of the infection. This so-called disease tolerance can protect against severe pathology. For example, tolerance to malaria has been linked to heme oxygenase-1 and to the iron sequestering protein ferritin^5,6^.

The adrenal cortex synthesizes glucocorticoids (GCs; mainly cortisol in humans and corticosterone in rats and mice) and mineralocorticoids. Adrenalin and noradrenalin are synthesized in the adrenal medulla. Together, these hormones regulate the homeostasis of critical physiological processes. GCs are produced in a circadian manner and upon activation of the hypothalamic pituitary adrenal (HPA) axis, during stress/trauma, infection or systemic inflammation^7^. They influence many processes ranging from metabolism, immunity, bone remodeling, cardiovascular function, reproduction and cognition^8^. GCs are well-known for their anti-inflammatory properties and have differential effects on various leukocyte subtypes^9,10^. In addition, gluconeogenesis, protein catabolism and lipolysis in, respectively, liver, muscle and adipose tissue contribute directly or indirectly to increased glucose levels in response to GCs^11^. Adrenalin is also produced as reaction to stress conditions to restore homeostasis, comprising the ‘fight-or-flight’ response.

Of the adrenal hormones, only GCs are increased upon human malaria infection. Blood cortisol levels are increased in *Plasmodium falciparum-* or *Plasmodium vivax*-infected patients, compared to healthy controls^12–14^. One study also showed higher cortisol levels in *P. falciparum-*infected patients with CM compared to uncomplicated malaria^15^. Despite the established findings of increased GC levels in patients infected with malaria, their functional role has received no recent attention. Studies in the 1980s concluded that in immunized mice, pregnancy-associated increases in corticosterone levels cause the loss of malarial immunity and more recrudescences^16–18^. An earlier and limited study reported that adrenalectomy reduced survival of NMRI mice after *P. berghei* K173-infection, though the mechanism was not explored^19^.

Here, adrenalectomy was performed to investigate the importance of adrenal hormones in experimental malaria. Several mouse-parasite strain combinations were used. Infection of C57BL/6 mice with *P. chabaudi* AS (*Pc*AS) is a model of resolving malaria with a non-lethal transient parasitemia accompanied by liver inflammation^20^. *P. berghei* NK65 Edinburgh strain (*Pb*NK65-E)-infected BALB/c mice represent a model of non-resolving malaria; the mice fail to clear the infection and die in a late phase due to hyperparasitemia and anemia^22^. In a model of hyperinflammatory malaria, infection of C57BL/6 mice with *Pb*NK65-E induces MA-ARDS, a lethal complication characterized by excessive lung inflammation^21^. Since important differences exist between malaria parasites that can infect normocytes versus parasites restricted to reticulocytes, we also included C57BL/6 mice infected with *Pb*NK65 New York strain (*Pb*NK65-NY), which predominantly infects reticulocytes^22^. Our data indicate that infection of adrenalectomized (Adx) mice leads to early lethal hypoglycemia and increased inflammation. The hypoglycemia is insulin- and TNF-α-independent and is paralleled by exhaustion of hepatic glycogen stores without increased gluconeogenesis. This phenotype can be rescued by dexamethasone (DEX) treatment. Therefore, adrenal hormones are essential for disease tolerance in malaria.

## Results

### Adrenal glands are crucial for disease tolerance in malaria

To investigate whether adrenal hormone production is essential to survive malaria infection, the adrenal glands were surgically removed from C57BL/6 and BALB/c mice, at least 16 days prior to infection. Sham-operated (Sham) mice were used as controls. C57BL/6 mice were infected with *Pb*NK65-E, *Pc*AS or *Pb*NK65-NY. BALB/c mice were also infected with *Pb*NK65-E. Adx mice showed no increase in plasma corticosterone levels upon infection, in contrast to Sham mice (Supplementary Fig. 1a,b). Non-infected control Adx and Sham mice did not show any clinical symptoms and survived the experiment.

**Fig. 1:**
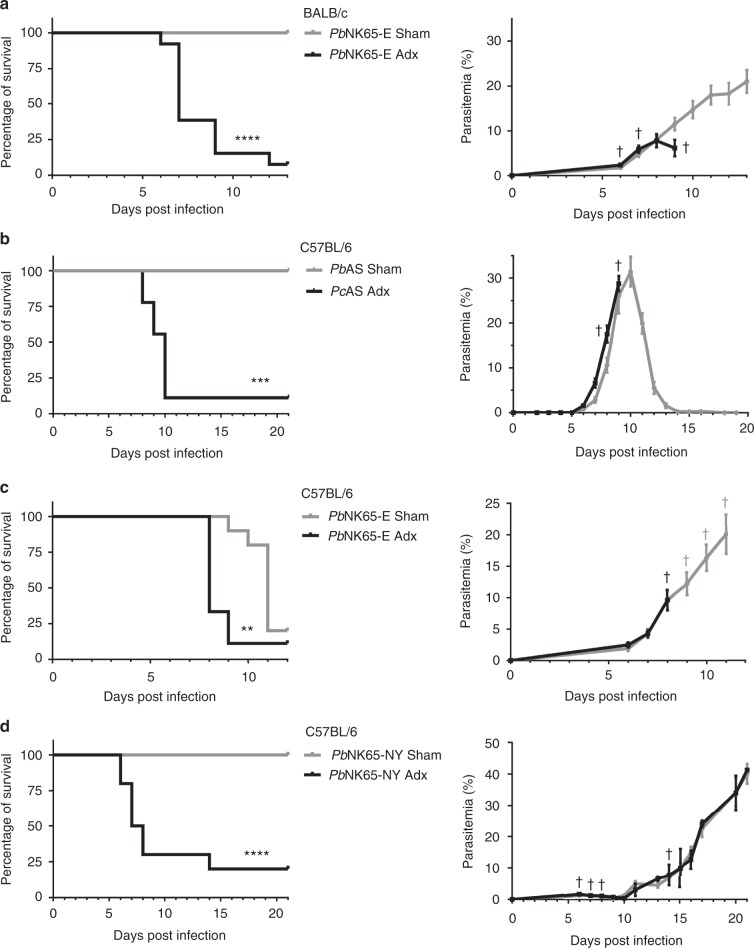
Adrenalectomy reduces survival following infection, without affecting parasitemia. Sham-operated (Sham) and adrenalectomised (Adx) BALB/c mice (**a**) or C57BL/6 mice (**b–d**) were injected with *Plasmodium berghei* NK65 Edinburgh strain (*Pb*NK65-E; **a** and **c**), *Plasmodium chabaudi* AS (*Pc*AS; **b**) or *Pb*NK65 New York strain (*Pb*NK65-NY; **d**). Survival of Adx and Sham mice was monitored daily. In each experiment, non-infected Sham and Adx mice were included and these all survived the observation period (not shown). The peripheral parasitemia of infected mice was determined at the indicated time points and shown as means ± SEM. Graphs represent two or three separate experiments and the numbers of mice are as follows: **a** Sham, *n* = 14; Adx, *n* = 13; **b** Sham, *n* = 8; Adx, *n* = 9; **c** Sham, *n* = 10; Adx, *n* = 9; **d** Sham, *n* = 12; Adx, *n* = 10. No parasitemia of Adx mice is shown where two or fewer mice remained alive: after day 9 (**a**, **b**) or day 8 (**c**). Daggers (†) indicate when at least one mouse died or was euthanized when it reached the humane endpoints. Asterisks indicate significance levels by Log-rank test. ** *p* < 0.01, *** *p* < 0.001, ****p < 0.0001

Importantly, early lethality was observed in the four mouse-parasite combinations, without affecting parasitemia. Infection of Adx BALB/c mice with *Pb*NK65-E caused lethality from 6 until 12 days post infection (p.i.) at a parasitemia of less than 10%, whereas the Sham-operated mice progressed later to hyperparasitemia and anemia (Fig. 1a). *Pc*AS-infected Adx C57BL/6 mice died between 8 and 10 days p.i., while the corresponding Sham mice all survived the transient parasitemia peak (Fig. 1b). *Pb*NK65-E-infected Adx C57BL/6 mice died 8 or 9 days p.i., whereas MA-ARDS in the Sham mice caused death around 11 days p.i. (Fig. 1c). Remarkably, although none of the Sham *Pb*NK65-NY-infected mice died before 21 days p.i, almost all *Pb*NK65-NY-infected Adx mice died 6–8 days p.i. at an average parasitemia as low as 1.6% (Fig. 1d).

Overall, this demonstrates, with four different animal models, that adrenalectomy does not influence parasitemia levels and that—regardless of the mouse-parasite combination—early mortality follows infection of Adx mice. Therefore, the adrenal glands are essential for disease tolerance in malaria.

To assess whether adrenalectomy affects other aspects of disease progression, body weight loss and disease severity were monitored. In all four animal models, the clinical disease score was higher in Adx mice upon infection compared to Sham mice (Supplementary Fig. 2, right panels). Adrenalectomy did not change body weight loss in *Pc*AS-infected mice, caused a slightly earlier body weight loss in *Pb*NK65-E-infected mice and significantly increased body weight loss in *Pb*NK65-NY-infected mice (Supplementary Fig. 2, left panels). Thus, adrenalectomy increases the disease severity following malaria infection and, dependent on the mouse-parasite combination, aggravates loss of body weight.

### Adrenalectomy does not affect peripheral pathology

In further experiments, we focused on *Pb*NK65-E-infected BALB/c and *Pc*AS-infected C57BL/6 mice, because these two models differ in mouse strain, parasite strain and resolving versus non-resolving infection in Sham mice. We investigated whether the adrenalectomy-induced mortality is related to malaria-associated anemia, liver damage, pulmonary pathology or kidney dysfunction. Therefore, Adx *Pb*NK65-E-infected BALB/c and *Pc*AS-infected C57BL/6 mice were examined when they had developed a clinical score of at least 3 (between 6 and 7 days p.i. for *Pb*NK65-E and 8 and 10 days p.i. for *Pc*AS), and were simultaneously compared to a corresponding number of infected Sham mice with similar parasitemia.

**Fig. 2:**
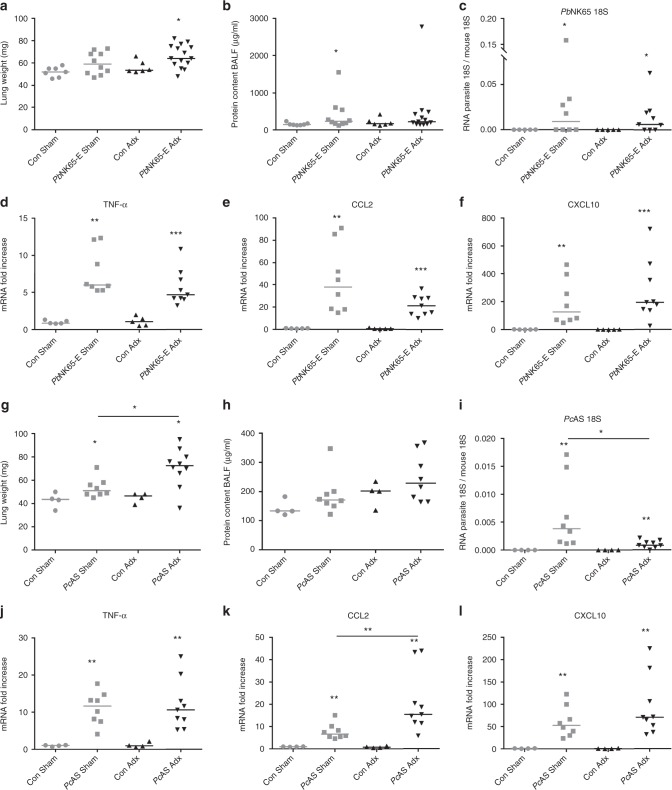
Adrenalectomy has no major effect on malaria-induced pathology and inflammation in the lungs. Sham and Adx BALB/c mice (**a**–**f**) or C57BL/6 mice (**g**–**l**) were infected with *Pb*NK65-E or *Pc*AS, respectively. Infected mice were euthanized and dissected at 6 to 7 days p.i. (*Pb*NK65-E) or 8 to 9 days p.i. (*Pc*AS). **a**, **g** Weight of left lungs. **b**, **h** Bronchoalveolar lavage fluid (BALF) samples were collected and the protein contents of centrifuged supernatants was determined. (**c**–**f** and **i**–**l**) Lungs were homogenized and specific mRNA levels were measured by qRT-PCR. Each symbol represents data from an individual mouse. Horizontal lines in between data points represent group medians and analysis was by Mann-Whitney *U*-test. Horizontal lines with asterisks on top indicate the levels of statistical significance between the indicated groups. Asterisks above individual data sets indicate the levels of statistical significance compared to the uninfected control group. Data from two separate experiments. * *p* < 0.05, ** *p* < 0.01, *** *p* < 0.001

Pulmonary pathology was determined by measuring the lung weight and protein content in the bronchoalveolar lavage fluid (BALF). Adrenalectomy did not affect lung weights in *Pb*NK65-E-infected Adx BALB/c compared to Sham mice (Fig. 2a), whereas an increase in lung weight was observed in *Pc*AS-infected Adx C57BL/6 compared to Sham mice (Fig. 2g). However, no difference was seen in protein content in the BALF between infected Adx and Sham mice, indicating that the mortality of Adx mice is not related to alveolar edema (Fig. 2b, h). Furthermore, in Adx mice, the accumulation of parasites in the lungs was comparable (Fig. 2c) or even lower than in Sham mice (Fig. 2i), despite similar peripheral parasitemia. Adrenalectomy did not consistently affect the pulmonary levels of mRNA encoding TNF-α, CCL2 (monocyte chemotactic protein-1; MCP-1) and CXCL10 (IFN-γ induced protein-10; IP-10) (Fig. 2d–f, j–l). Nevertheless, the increase in pulmonary CCL2 expression in Adx mice upon *Pc*AS infection was significantly greater than that in Sham mice (Fig. 2k).

Liver pathology was assessed by measurement of markers of liver damage. Infection with *Pc*AS, but not with *Pb*NK65-E, increased plasma alanine aminotransferase (ALT) levels in Adx mice (around 250 IU/L). This suggests greater liver damage in Adx compared to Sham mice after *Pc*AS infection (Supplementary Fig. 3a,b). However, these increased ALT levels are unlikely to indicate liver damage that is sufficient to cause an abrupt death, since non-operated *Pc*AS-infected C57BL/6 mice exhibit tenfold higher ALT levels (up to 3000 IU/L) at a later time point without any lethality^20^. Accumulation of *Pc*AS parasites, which are known to sequester in the liver, was lower in infected Adx mice (Supplementary Fig. 3c). To investigate whether death of infected Adx mice was associated with increased hepatic pro-inflammatory cytokine expression, several mRNA transcripts were measured in the liver. In accordance with previous observations^20^, infection with *Pc*AS increased hepatic levels of several mRNAs encoding pro-inflammatory cytokines. No significant differences in levels of mRNAs encoding IL-1β or IL-6 were seen between Adx and Sham mice (Supplementary Fig. 3f,g). CCL2 and TNF-α mRNA levels were even lower in Adx than in Sham mice (Supplementary Fig. 3d,e). This reinforces the notion that liver inflammation is not the cause of death in the *Pc*AS-infected Adx mice.

Although hemoglobin levels were decreased upon infection, no differences between Adx and Sham mice were observed (Supplementary Fig. 3h). Furthermore, kidney function, assessed by urinary albumin/creatinine ratio, was similar between groups (Supplementary Fig. 3i).

Altogether, these data indicate that adrenalectomy does not affect parasite growth or accumulation, nor does it affect anemia, liver, lung or kidney function. The observed lethality must therefore be due to other causes.

### Adrenalectomy increases brain inflammation upon infection

**Fig. 3:**
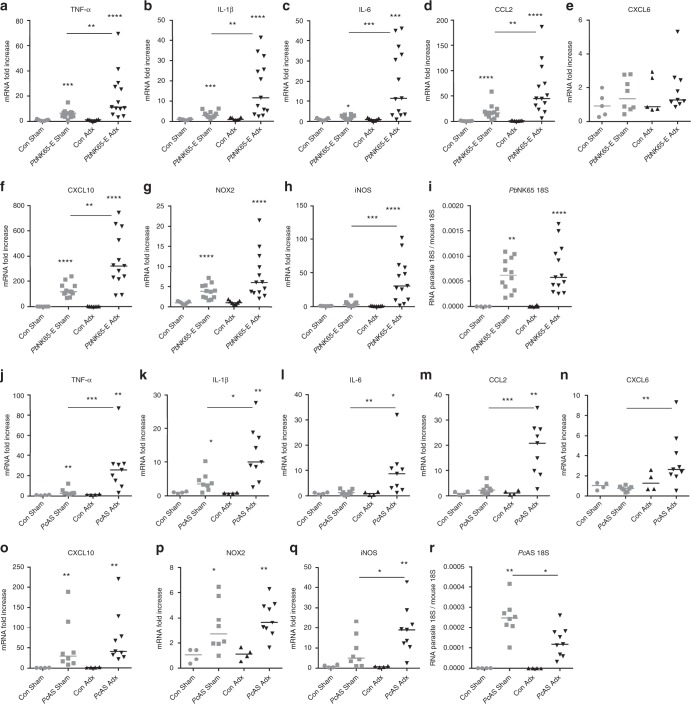
Pro-inflammatory cytokine and chemokine expression is increased in the brain of Adx mice upon infection. Sham and Adx BALB/c mice or C57BL/6 mice were infected with *Pb*NK65-E (**a**–**i**) or *Pc*AS (**j**–**r**), respectively. Infected mice were euthanized and dissected at 6 to 7 days p.i. (*Pb*NK65-E) or 8 to 9 days p.i. (*Pc*AS). Brains were homogenized and specific mRNA levels were measured by qRT-PCR. Each symbol represents data from an individual mouse. Horizontal lines in between data points represent group medians and analysis was by Mann-Whitney *U*-test. Horizontal lines with asterisks on top indicate the levels of statistical significance between the indicated groups. Asterisks above individual data sets indicate the levels of statistical significance compared to the uninfected control group. Data from at least two separate experiments. * *p* < 0.05, ** *p* < 0.01, *** *p* < 0.001, ****p < 0.0001

In the experimental malaria models used, neurological symptoms developed in infected Adx mice (Supplementary Movie 1). Therefore, levels of mRNA transcripts encoding inflammatory cytokines and chemokines were measured in the brain (Fig. 3). Notably, the upregulation of the pro-inflammatory TNF-α, IL-1β, IL-6 and CCL2 was significantly higher in *Pb*NK65-E and *Pc*AS-infected Adx compared to Sham mice (Fig. 3a–d, j–m). In general, mRNAs encoding the chemokines CXCL6 (granulocyte chemotactic protein-2; GCP-2) and CXCL10 were differentially upregulated in *Pb*NK65-E and *Pc*AS-infected Adx compared to Sham mice (Fig. 3e, f, n, o). Furthermore, the levels of mRNA encoding the nitric oxide-producing enzyme, inducible nitric oxide synthase (iNOS), showed a higher increase in Adx compared to Sham mice upon *Pb*NK65-E and *Pc*AS infection (Fig. 3h, q), whereas the expression of reactive oxygen producing NADPH oxidase 2 (NOX-2) was not affected (Fig. 3g, p). The higher expression of cytokines, chemokines and iNOS in brains of Adx mice was not the consequence of greater parasite accumulation in the brain, as levels of parasite 18S RNA were either similar (in *Pb*NK65-E-infected BALB/c; Fig. 3i) or lower (in *Pc*AS-infected C57BL/6; Fig. 3r) in Adx compared to Sham mice.

**Fig. 4:**
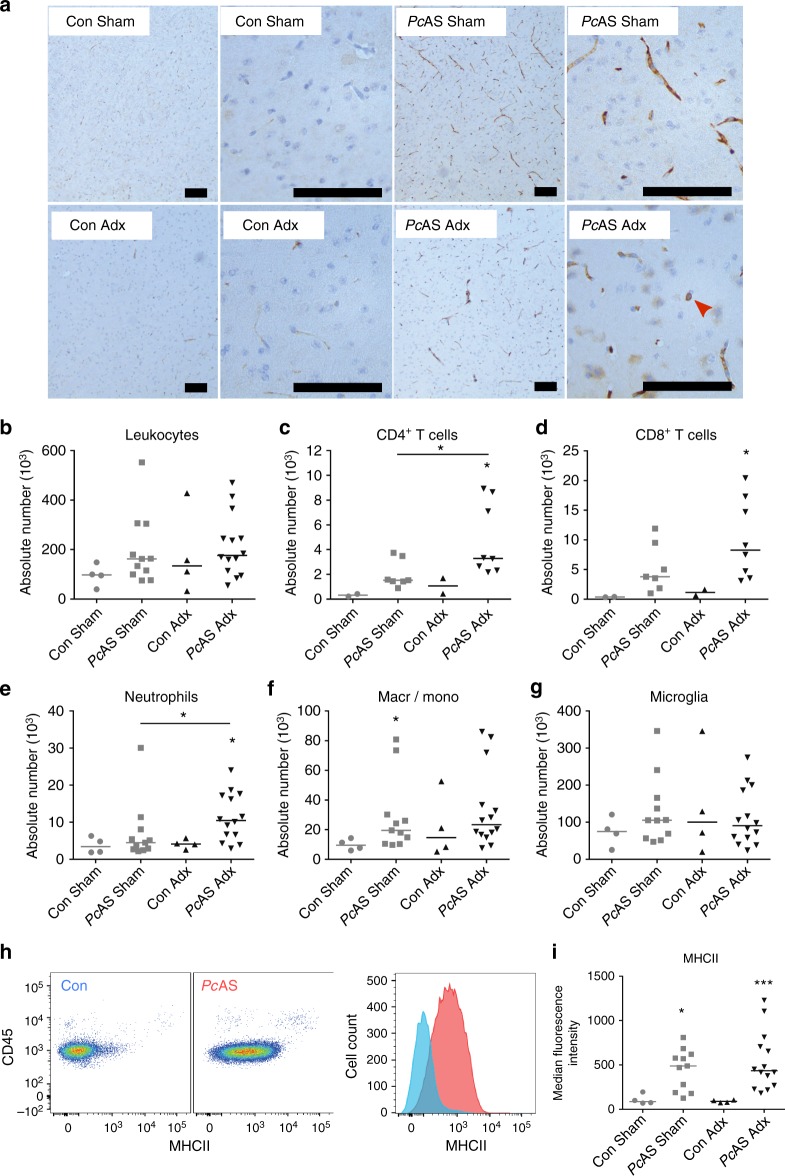
CD4^+^ T cell and neutrophil numbers are increased in brains of Adx mice upon infection. Sham and Adx C57BL/6 mice were infected with *Pc*AS. The mice were euthanized and brains were dissected at 8 to 10 days after infection. **a** Sections were stained with anti-CD45 monoclonal antibodies (brown) to identify leukocytes. Representative images are shown (original magnification x10 and x40, bar = 100 µm). The red arrow indicates an occasional leukocyte. **b**–**i** Leukocytes were isolated from individual brains and analyzed by flow cytometry to identify CD45^+^ leukocytes. **c**–**f** Tmem119^−^ cells were subdivided into CD4^+^ T cells (CD3^+^CD4^+^), CD8^+^ T cells (CD3^+^CD8^+^), neutrophils (CD11b^+^Ly6G^+^F4/80^−^) and macrophages/monocytes (CD11b^+^F4/80^+^). **g** Microglia were gated as CD11b^+^Tmem119^+^Ly6G^−^CD3^−^. **h**–**i** Characterization of MHC class II expression on microglia. **h** Plots representing the staining pattern for the indicated markers. Corresponding intensity of MHC class II expression on microglia from a representative control mouse (blue) and a *Pc*AS infected mouse (red). **i** Median fluorescence intensity of MHC class II. Each symbol represents data from an individual mouse. Horizontal lines in between data points represent group medians and analysis was by Mann-Whitney *U*-test. Horizontal lines with asterisks on top indicate the levels of statistical significance between the indicated groups. Asterisks above individual data sets indicate the levels of statistical significance compared to the uninfected control group. Data from two separate experiments. * *p* < 0.05, ** *p* < 0.01, *** *p* < 0.001

To investigate leukocyte infiltration of the brain, immunohistochemistry was performed with an anti-CD45 antibody on sagittal brain sections of *Pc*AS-infected C57Bl/6 mice (Fig. 4a). More intense immunoreactivity was observed in infected mice compared to uninfected controls. However, no major differences were found between Adx and Sham mice in any region of the brain. Therefore, flow cytometry was performed to quantify leukocytes in the brain (Fig. 4b–i and Supplementary Fig. 4). Amongst the infiltrating cell populations, numbers of CD4^+^ T lymphocytes and neutrophils were greater in Adx versus Sham mice (Fig. 4c, e). The infection did not alter the number of microglia, as identified by the specific Tmem119 marker^23^ (Fig. 4g), but increased their activation state, as determined by elevated MHC class II expression levels (Fig. 4h, i). However, activation did not differ between Adx and Sham infected mice. Together, the increased cerebral cytokine expression levels and numbers of locally recruited neutrophils and CD4^+^ T cells indicate that malaria infection leads to an increased inflammatory activity in the brain of Adx compared to Sham mice.

### Plasma cytokines are elevated in *Pc*AS-infected Adx mice

Adrenalectomy enhances endotoxin-induced lethality related to increased circulating cytokines^24^. Hence, we assessed the impact of adrenalectomy on plasma cytokine and chemokine levels following infection with *Pc*AS. Plasma levels of TNF-α, IFN-γ, IL-6, IL-10, CXCL10, CCL2 and IL-4 were all increased upon infection (Supplementary Fig. 5). Of these, TNF-α, IFN-γ, IL-6 and IL-10 were elevated to a greater extent in Adx compared to Sham mice. The expression of TNF-α, IFN-γ, IL-6, IL-10 and IL-1β was also examined in the spleen (Supplementary Fig. 6). Only the levels of mRNAs encoding IL-6 and IL-1β were increased in spleens of infected Adx compared to Sham mice, while the level of the mRNA encoding TNF-α was even decreased.

In summary, these data indicate that the severe pathology in infected Adx mice is associated with raised levels of cytokines, both in the brain and in the circulation. Notably, adrenal hormones differentially affect inflammation in the brain and circulation versus the liver and lungs.

### Lethal hypoglycemia develops in infected Adx mice

**Fig. 5:**
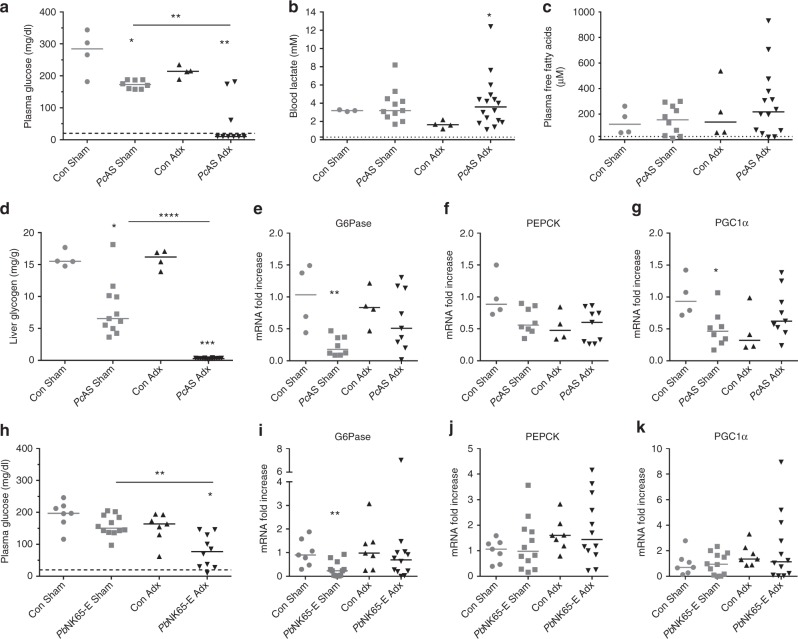
Severe hypoglycemia and depleted glycogen stores without alterations in lactate, free fatty acids and hepatic gluconeogenic enzyme expression in infected Adx mice. Sham and Adx C57BL/6 or BALB/c mice were infected with *Pc*AS (**a**–**g**) or *Pb*NK65-E (**h**–**k**), respectively. Infected mice were euthanized and dissected at 8 to 10 days p.i. (*Pc*AS) or 6 to 7 days p.i (*Pb*NK65-E). **a**,**h** Glucose levels were determined in plasma (detection limit was 20 mg/dL, indicated by dotted line). Samples with a measurement below the detection limit, were given an arbitrary value of 10 mg/dL. **b**, **c** Blood lactate and plasma free fatty acid levels were measured. The dotted lines represent the limits of detection. **d** Hepatic glycogen content (in mg/g of tissue). (**e**–**g**, **i**–**k**) Livers were homogenized and specific mRNA levels were measured by qRT-PCR. Each symbol represents data from an individual mouse, (**d**) *n* = 14 for *Pc*AS Adx. Horizontal lines in between data points represent group medians and analysis was by Mann-Whitney *U*-test. Horizontal lines with asterisks on top indicate the levels of statistical significance between the indicated groups. Asterisks above individual data sets indicate the levels of statistical significance compared to the uninfected control group. Data from at least two separate experiments. * *p* < 0.05, ** *p* < 0.01, *** *p* < 0.001, ****p < 0.0001

Since GCs and adrenalin have glucose-regulating properties, and malaria may be complicated by hypoglycemia, the effect of adrenalectomy on blood glucose levels was assessed. In general, glucose levels decreased after infection (although not significantly for *Pb*NK65-E-infected Sham BALB/c mice; Fig. 5a, h). However, the drop in plasma glucose levels was considerably greater in infected Adx mice compared to infected Sham mice. The hypoglycemia was most marked in *Pc*AS-infected Adx mice. To determine how generally applicable this finding was, glycemia was also measured during the course of infection of Adx and Sham C57BL/6 mice with *Pb*NK65-E or *Pb*NK65-NY (Supplementary Fig. 7). In these two models, severe hypoglycemia developed within 9 days after infection. This marked hypoglycemia was sufficient to cause death, as plasma glucose levels below 50 mg/dL cause functional brain failure and coma, and levels below 20 mg/dL result in rapid brain death^25^.

Only in *Pb*NK65-E-infected C57BL/6 mice, glucose levels correlated negatively with parasitemia (Supplementary Fig. 7b), but not in the other three models (Supplementary Fig. 7f and Supplementary Fig. 8a-b). Also, although glycemia correlated negatively with plasma ALT levels after infection with *Pc*AS (Supplementary Fig. 8d), no correlation was observed after infection with *Pb*NK65-E (Supplementary Fig. 8c). Consistent with hypoglycemia being a major life threat, glycemia correlated negatively with the clinical score (Supplementary Fig. 7d, h). Furthermore, plasma glucose levels correlated negatively with brain levels of mRNAs encoding TNF-α, IL-1β, IL-6, CCL2 and iNOS, suggesting that hypoglycemia and the expression of these pro-inflammatory markers might be linked (Supplementary Fig. 8e–n). With the exception of IL-4, cytokine and chemokine concentrations in the plasma also correlated negatively with plasma glucose levels (Supplementary Fig. 8o–u).

Besides hypoglycemia, hyperlactatemia and lactic acidosis may complicate malaria infections and may result from an increased glycolytic flux. Blood lactate levels were increased in Adx mice upon infection but were not different from Sham infected mice (Fig. 5b). A modest negative correlation was found with the blood glucose levels (Supplementary Fig. 8×).

As an alternative energy source for glucose, plasma levels of free fatty acids (FFA) are increased after stimulation of lipolysis in adipose tissue by adrenalin and GCs. Nevertheless, plasma FFA levels were not decreased in *Pc*AS-infected Adx compared to Sham mice (Fig. 5c), indicating that limiting levels of FFA were not driving the hypoglycemia.

A deficiency in glycogenolysis as well as an insufficient gluconeogenic response might lead to hypoglycemia. Therefore, glycogen levels were measured in liver extracts (Fig. 5d). Glycogen stores decreased upon infection of Sham mice and were fully depleted in infected Adx mice, suggesting that hepatic glycogen was consumed and exhausted during the infection of Adx mice. In order to investigate possible alterations in hepatic gluconeogenesis, levels of mRNAs encoding the two main gluconeogenic flux-controlling enzymes, glucose-6-phosphatase (G6Pase) and phosphoenolpyruvate carboxykinase (PEPCK) were measured. Expression did not differ between Adx and Sham C57BL/6 mice or BALB/c mice upon infection with either *Pc*AS (Fig. 5e, f) or *Pb*NK65-E (Fig. 5i, j), respectively. Remarkably, despite the increased corticosterone levels in Sham mice upon infection, expression of PEPCK was not induced and G6Pase expression was even decreased after infection. We also explored the hepatic expression of peroxisome proliferator-activated receptor gamma coactivator 1-alpha (PGC1α), a GC-inducible transcriptional coactivator which coordinates the expression of a wide array of genes involved in glucose and fatty acid metabolism. Surprisingly, neither infection, nor adrenalectomy affected the expression of PGC1α (Fig. 5g, k). These data thus indicate that the hepatic gluconeogenic transcriptional response is diminished after malaria infection, despite increased GC levels.

Altogether, these findings point to adrenal hormones being crucial in malaria infections to maintain blood glucose levels. In Adx mice, hepatic glycogen was completely exhausted and thus unable to prevent the development of severe hypoglycemia. The classical glycemia-regulating mechanisms of adrenal hormones include the transcriptional induction of a gluconeogenic response in the liver and the increase of FFA plasma levels. However, this did not occur upon malaria infection suggesting that adrenal hormones confer protection against hypoglycemia through other mechanisms.

### Glucose supplementation fails to rescue infected Adx mice

**Fig. 6:**
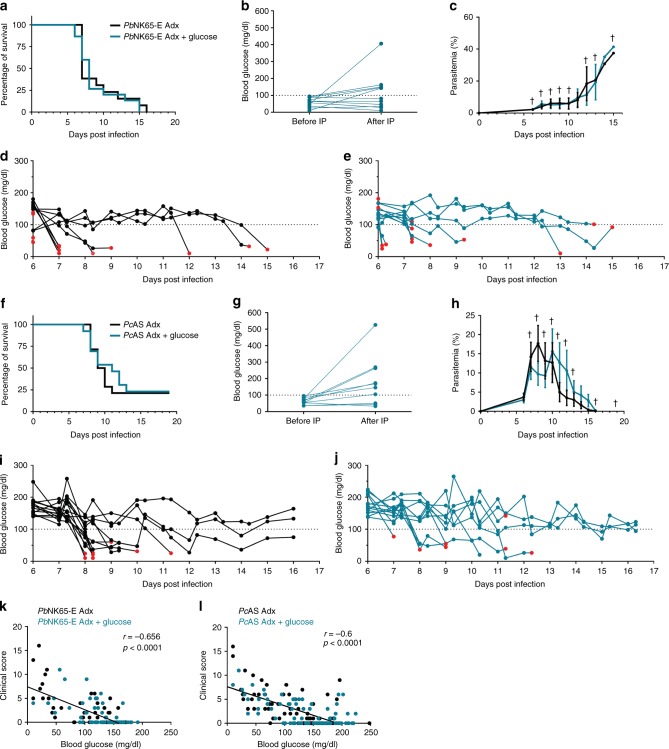
Supplementation with glucose does not rescue from hypoglycemia or lethality in infected Adx mice. Adx BALB/c mice mice were infected with *Pb*NK65-E (**a**–**e** and **k**) and Adx C57BL/6 mice with *Pc*AS (**f**–**j** and **l**). Glucose supplementation was started 5 days p.i. via the drinking water (5% (wt/vol)) and by IP injection of a dose of 2 g/kg if the blood glucose levels fell below 100 mg/dL (dotted lines). In each experiment, non-infected Adx mice were included and these all survived the observation period (not shown). **a, f** Survival of the mice was monitored daily. **b**, **g** Blood glucose levels were measured before and 30 min after the first IP injection of glucose. **c**, **h** The peripheral parasitemia of infected mice was determined at the indicated time points. Data are means ± SEM. Daggers (†) indicate when at least one mouse died or was euthanized when it reached the humane endpoints. **d**, **e** and **i**, **j** Morning and afternoon blood glucose levels were measured. Each curve represents the glycemia course of an individual mouse, black curves (**d**, **i**) for untreated mice and blue curves (**e, j**) for glucose-supplemented mice. Red dots indicate the last glucose measurement before death. **k, l** Spearman correlations between blood glucose levels of both glucose-supplemented and untreated mice and the clinical score. Spearman *r*- and *p*-values are shown. Graphs represent two separate experiments and the numbers of mice are as follows: *n* = 4 for uninfected controls, *n* > 10 for infected Adx without or with glucose

To test whether glucose supplementation could alleviate the severe hypoglycemia, *Pc*AS- or *Pb*NK65-E-infected Adx mice were provided with drinking water supplemented with glucose (5% wt/vol) and additionally, if blood glucose levels fell below 100 mg/dL, an intraperitoneal (IP) injection of 2 g/kg. Remarkably, glucose supplementation did not prevent the lethal effect of infection in Adx mice (Fig. 6a, f) nor was it sufficient to prevent or reverse the hypoglycemia (Fig. 6d, e, i, j). In only 18% (2/11) or 40% (4/10) of the *Pb*NK65-E or *Pc*AS-infected Adx mice, respectively, the first IP injection was able to increase blood glucose levels with 100 mg/dL in 30 min (Fig. 6b, g). The parasitemia was unaffected by glucose administration (Fig. 6c, h). With or without glucose supplementation, the blood glucose levels correlated significantly with the clinical score (Fig. 6k, l), in line with earlier results (Supplementary Fig. 7d,h).

### Adrenalectomy-induced hypoglycemia is not caused by insulin

**Fig. 7:**
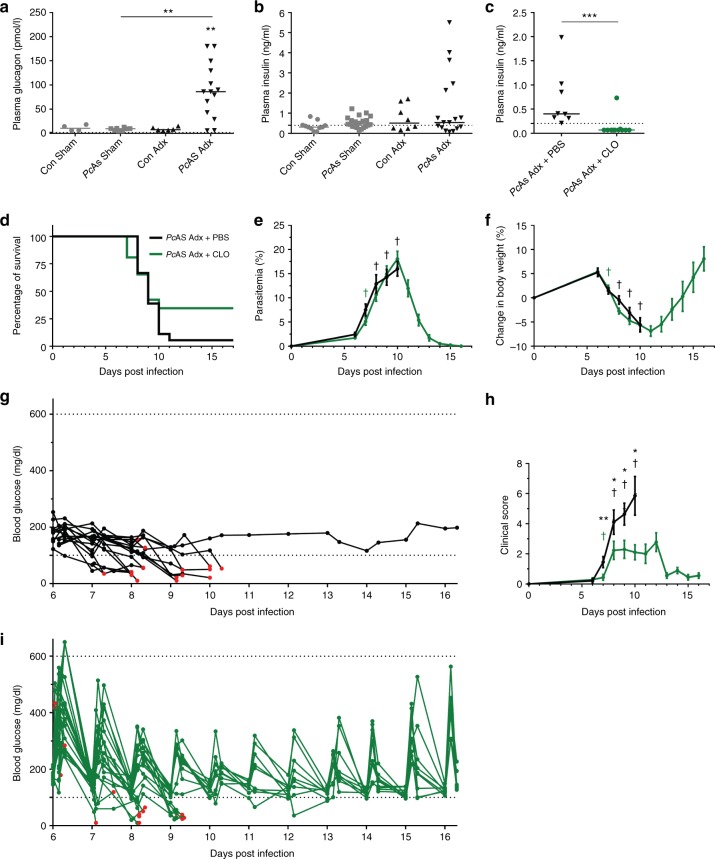
Inhibition of insulin secretion by clonidine cannot revert lethal hypoglycemia in PcAS-infected Adx mice. **a**, **b** Sham and Adx C57BL/6 mice were infected with *Pc*AS. Mice were euthanized and dissected at 8 to 10 days p.i. and plasma glucagon and insulin levels were measured. **c**–**i** Adx C57BL/6 mice were infected with *Pc*AS. Daily clonidine (CLO) treatment was started at 6 days p.i. by IP injection of a dose of 0.5 mg/kg or 1 mg/kg clonidine hydrochloride dissolved in PBS if glycemia was, respectively, above or below 100 mg/dL. Control mice were treated with the corresponding volume of PBS. **c** Plasma insulin levels at 8 to 10 days after infection. The dotted lines represent the limits of detection. Each symbol represents data from an individual mouse, with *n* = 11 for *Pc*AS Adx + CLO. Horizontal lines in between data points represent group medians and analysis was by Mann-Whitney *U*-test. Horizontal lines with asterisks on top indicate the levels of statistical significance between the indicated groups. Asterisks above individual data sets indicate the levels of statistical significance compared to the uninfected control group. **d** Survival of mice was monitored daily. **e**, **f**, **h** The peripheral parasitemia, relative changes in body weight compared to day 0 and clinical scores were determined at the indicated time points. Data are means ± SEM and analysis was by Mann-Whitney *U*-test. Asterisks on top indicate a statistically significant difference between treated and untreated mice. Daggers (†) indicate when at least one mouse died or was euthanized when it reached humane endpoints. No data are shown for Adx + PBS after 10 days p.i. because two or fewer mice remained alive. **g**, **i** Blood glucose levels were measured in the morning, 1 h after clonidine injection and in the afternoon. Each curve represents the glycemia course of an individual mouse, black curves (**g**) for untreated mice and green curves (**i**) for treated mice. Red dots indicate the last glucose measurement before death. Graphs represent at least 4 separate experiments and the numbers of mice are as follows: *n* > 17 for Adx + PBS, *n* > 25 for Adx + CLO. * *p* < 0.05, ** *p* < 0.01, *** *p* < 0.001

Insulin and glucagon are essential pancreatic hormones that regulate glycemia. Plasma glucagon levels were highly increased in infected Adx mice with hypoglycemia (Fig. 7a and Supplementary Fig 8w), suggesting that pancreatic α-cells reacted correctly to the low glycemia. Plasma insulin levels were not increased in *Pc*AS-infected Adx mice compared to Sham mice, but were not suppressed either, despite the severe hypoglycemia (Fig. 7b). In fact, insulin levels did not correlate with glucose levels (Supplementary Fig. 8v). Since the administered glucose was rapidly cleared from the circulation (Fig. 6) and insulin levels were not decreased in the hypoglycemic infected Adx mice, we tested whether suppression of insulin release could alleviate the hypoglycemia. *Pc*AS-infected Adx mice were treated with clonidine (Fig. 7c-i), an α2-adrenergic agonist, because it is well documented that α2-adrenergic agonism inhibits insulin-secretion from β-cells of the pancreas^26,27^. Clonidine treatment indeed drastically decreased plasma insulin levels (Fig. 7c). In mice with glycemia levels above 100 mg/dL, this resulted in marked but transient increases in glycemia (Fig. 7i). The average clinical score in the clonidine-treated mice was also lower (Fig. 7h). However, clonidine treatment did not significantly improve the survival of the mice (Fig. 7d) and was, despite dramatically decreased insulin levels, unable to restore or increase glycemia in most of the mice that developed hypoglycemia (Fig. 7i). Parasitemia or weight loss also did not differ (Fig. 7e, f). Together, these data indicate that inappropriate release of insulin is not the driver of hypoglycemia in the Adx mice.

### TNF-α neutralization is not sufficient to prevent lethality

**Fig. 8:**
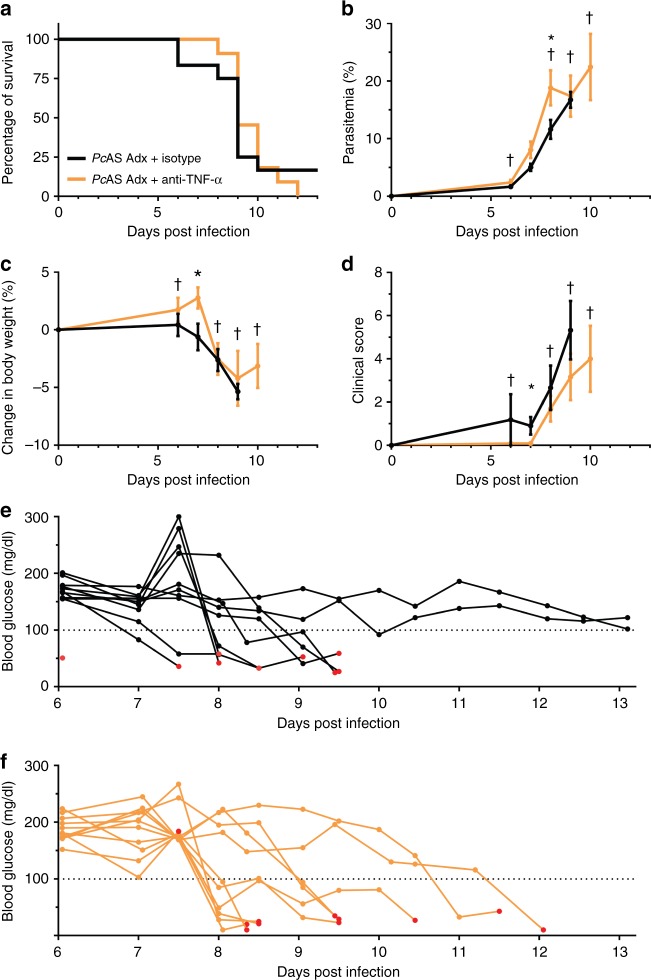
Neutralization of TNF-α does not affect the disease course and survival of infected Adx mice. Adx C57BL/6 mice were infected with *Pc*AS. TNF-α was neutralized by IP injection of 0.5 mg anti-TNF-α antibody at 5, 8 and 11 days after infection. Control mice were treated with the corresponding monoclonal antibody isotype control. **a** Survival of mice was monitored daily. **b**–**d** The peripheral parasitemia, relative changes in body weight compared to day 0 and clinical scores were determined at the indicated time points. Data are means ± SEM and analysis was by Mann-Whitney *U*-test. Asterisks on top indicate statistically significant differences between treated and untreated mice. Daggers (†) indicate when at least one mouse died or was euthanized when it reached the humane endpoints. No data are shown after 9 days and 10 days p.i. for, respectively, Adx + Isotype and Adx + anti-TNF-α because 2 or fewer mice remained alive. **e**, **f** Blood glucose levels were measured in the morning and in the afternoon. Each curve represents the glycemia course of an individual mouse, black curves (**e**) for untreated mice and orange curves (**f**) for treated mice. Red dots indicate the last glucose measurement before death. Graphs represent two separate experiments and the numbers of mice are as follows: *n* = 12 for Adx + Isotype, *n* = 11 for Adx + anti-TNF-α. * *p* < 0.05

In the infected Adx mice, TNF-α was one of the most increased cytokines in the circulation (Supplementary Fig. 5) and brain (Fig. 3). Since, amongst the increased cytokines, this was the main cytokine able to cause acute lethality, we performed TNF-α neutralization experiments with a monoclonal antibody in *Pc*AS-infected Adx mice. Survival was unaltered by TNF-α neutralization (Fig. 8a). Moreover, parasitemia, body weight loss and clinical score were only minimally affected (Fig. 8b–d). The *Pc*AS-infected Adx mice still developed lethal hypoglycemia following TNF-α neutralization (Fig. 8f). These findings indicate that suppression of TNF-α is not sufficient to protect against lethal hypoglycemia.

### Dexamethasone treatment reverses the lethal phenotype

**Fig. 9:**
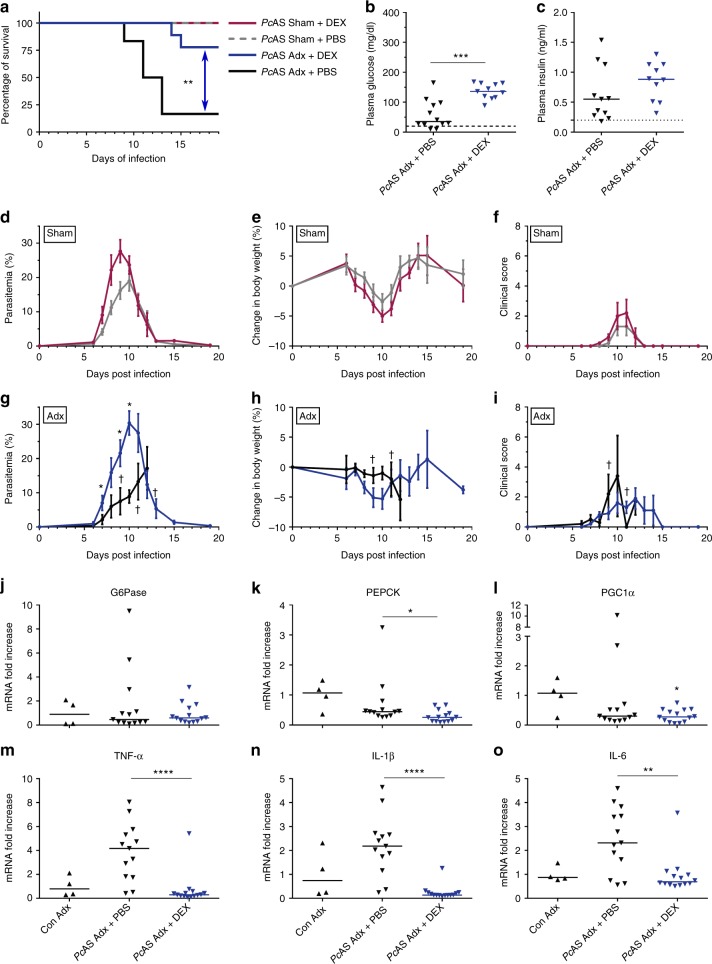
Dexamethasone treatment of Adx mice prevents the lethal hypoglycemia and suppresses cerebral cytokines upon infection. Sham and Adx C57BL/6 mice were infected with *Pc*AS. Dexamethasone (DEX) treatment was started 4 to 5 days p.i. by IP injection of a dose of 3 mg/kg dexamethasone sodium phosphate dissolved in PBS. Control mice were treated with the corresponding volume of PBS. **a** Survival of Adx and Sham mice was monitored daily. Two infected and treated Adx mice were euthanized after the peak in parasitemia on day 14 and 15 p.i. because of DEX side-effects. Asterisks indicate significance levels by Log-rank test. **b**, **c** Glucose and insulin levels were determined in plasma at day 8 to 10 p.i (detection limits indicated by dotted line). **d**, **g** The peripheral parasitemia was determined at the indicated time points. **e**, **h** The relative changes in body weight, compared to day 0, were determined at the indicated time points. **f**, **i** A clinical score of the disease severity was calculated based on several clinical parameters. **j–o** At day 8 to 10 p.i., mice were euthanized, liver (**j**–**l**) and brain (**m**–**o**) were homogenized and specific mRNA levels were measured by qRT-PCR. **d**–**i** Data are means ± SEM and analysis was by Mann-Whitney U-test. Daggers (†) indicate when at least one mouse died or was euthanized when it reached the humane endpoints. No data are shown for Adx + PBS after day 12 when two or fewer mice remained alive. Graphs represent two separate experiments and the numbers of mice are as follows: *n* = 5 for infected Sham + DEX, *n* = 6 for infected Sham + PBS, *n* = 9 for infected Adx + DEX, *n* = 6 for infected Adx + PBS. **b**, **c**, **j**–**o** Each symbol represents data from an individual mouse. Horizontal lines in between data points represent group medians and analysis was by Mann-Whitney *U*-test. Horizontal lines with asterisks on top indicate the levels of statistical significance between the indicated groups. Asterisks above individual data sets indicate the levels of statistical significance compared to the uninfected control group. * *p* < 0.05, ** *p* < 0.01, *** *p* < 0.001, ****p < 0.0001

We evaluated whether the lethal phenotype of *Pc*AS-infected Adx C57BL/6 mice could be rescued by treatment with a synthetic GC, DEX. An IP injection of 3 mg/kg DEX was administered daily to *Pc*AS-infected Adx and Sham mice, from 4 or 5 days after infection. DEX improved the survival of Adx *Pc*AS-infected mice (Fig. 9a) despite higher levels of parasitemia in treated compared to untreated mice (Fig. 9d, g). Due to DEX side-effects (ascites and gastro-intestinal complications) two out of nine infected Adx mice had to be euthanized on day 14 or day 15 p.i., after their peak in parasitemia. Following infection, DEX treatment did not alter body weight loss or clinical score (Fig. 9e, f, h, i). Importantly, DEX increased plasma glucose levels in Adx mice at their peak of parasitemia, 8 to 10 days p.i. (Fig. 9b), without significantly affecting insulin levels (Fig. 9c). Surprisingly, the levels of mRNAs encoding G6Pase, PEPCK and PGC1α were not induced in the liver upon DEX treatment (Fig. 9j-l). However, DEX did potently suppress the inflammatory cytokine expression in the brain (Fig. 9m–o).

Together, these data show that the hypoglycemia in infected Adx mice is not reversed by administration of glucose, by blocking insulin release with clonidine or by neutralizing TNF-α. In contrast, DEX treatment is able to increase the glycemia, to suppress cerebral cytokine expression and to prevent lethality.

## Discussion

**Fig. 10:**
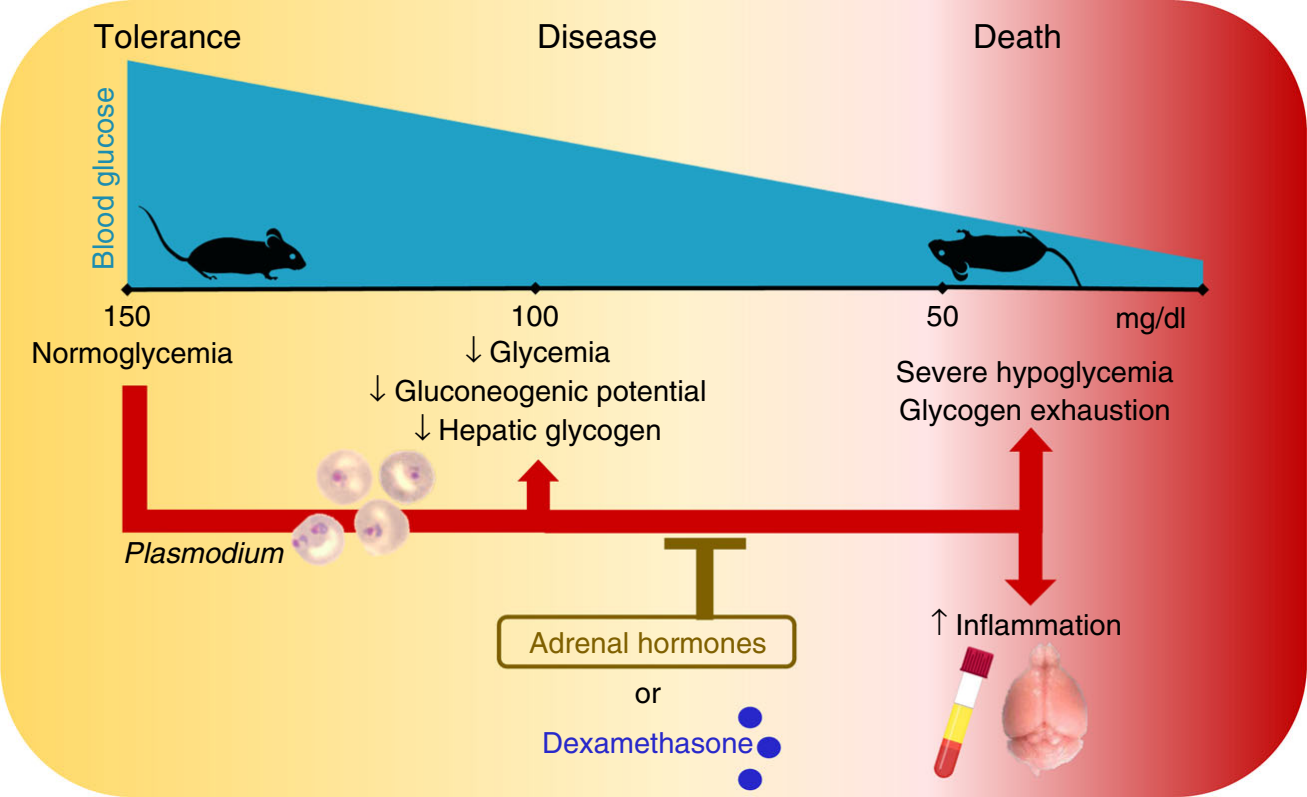
Adrenal hormones confer tolerance to malarial disease. Blood glucose levels decrease slightly, hepatic glycogen is consumed and the expression of a main gluconeogenic enzyme, glucose-6-phosphatase is lowered in murine malaria models without affecting survival. However, when adrenal hormone production is abolished, glycogen stores become exhausted and glycemia declines further until lethal glycemia levels are reached. This hypoglycemia is accompanied by increased circulatory and cerebral cytokine levels and can be prevented by supplementing dexamethasone. These effects are independent of the parasite load, indicating that adrenal hormones are crucial in mediating tolerance to malarial disease

In this study, we demonstrated that adrenal hormones confer disease tolerance to murine malaria (Fig. 10). Without affecting the pathogen load, adrenal hormones prevent the development of severe hypoglycemia and suppress cytokine levels in the circulation and brain but, surprisingly, not in the lungs or liver. Treatment with the synthetic glucocorticoid DEX compensates for the adrenalectomy after *Pc*AS infection, indicating that GCs can restore tolerance to malaria.

Our data show the crucial role of adrenal hormones in four different mouse-parasite combinations, which mimic different outcomes of malaria. This indicates the general applicability of our findings in murine malaria. It is striking that in the model with C57BL/6 mice infected with *Pb*NK65-NY, adrenalectomy resulted in death at low parasitemia (on average 1.5% infected red blood cells), levels at which the Sham mice did not display any symptoms. This suggests that adrenal hormones are essential for disease tolerance in asymptomatic malaria. Although it is currently unclear whether these findings may be extrapolated to humans, possibly it is of importance, since hundreds of millions of people are asymptomatically infected with malaria^28^. A report of adrenocortical and pituitary hyporesponsiveness in patients with complicated malaria suggest that HPA axis dysregulation might contribute to disease severity^12^.

Previously, the notion of disease tolerance has been applied to malaria and other infection models. In malaria, tolerance mechanisms have been ascribed to heme oxygenase-1, carbon monoxide, ferritin and nitric oxide, though these mechanisms are likely to be interlinked^5,29,30^. In the absence of one of these mediators, the host loses its capacity to survive the infection, irrespectively of the parasitemia. As a new tolerance mechanism in malaria, we here show the importance of adrenal hormones, which are essential to maintain a minimal glycemia.

Tolerance mechanisms have also been identified in bacterial and viral infections and largely reflect metabolic adaptations of the host. In an innovative study, Weis et al. linked the ability of ferritin to prevent hypoglycemia with tolerance to sepsis^31^. This was mediated by sustained expression of liver G6Pase. In another elegant study, Wang et al. reported opposing effects of fasting on tissue tolerance in bacterial versus viral infection^32^. Fasting dampened bacterial inflammation since glucose stimulated brain damage, whereas the outcome of a viral infection was favored by glucose through attenuation of brain dysfunction.

The host nutritional status and metabolism influence the immune function and outcome of infection, potentially affecting pathogen clearance. In experimental CM, early dietary restriction protects against neuropathology^33^. This is associated with an increased capacity of the spleen to clear parasitized red blood cells (RBCs). These effects have been attributed to decreased levels of leptin, a host-derived adipokine, which activates mTORC1 in splenic T cells. Conversely, a high-fat diet mediates oxidative stress in hepatocytes, preventing *Plasmodium* survival inside hepatocytes^34^. Cumnock et al. propose that, in the *P. chabaudi chabaudi* AJ murine model of malaria, the combination of anorexia and severe anemia makes the host reliant upon glycolysis. This is supported by the lethal effect of 2-deoxyglucose and the improved survival after glucose supplementation^35^. Furthermore, circadian rhythms of food intake dictate the synchronicity of the erythrocytic stage of *Pc*AS^36,37^. Many studies addressing the relevance of metabolism to malaria focus on the metabolism of the parasite^38–40^. Importantly, metabolic changes in patients with malaria contribute considerably to the severity of disease. Lactate acidosis and hypoglycemia are key indicators of a poor prognosis^41,42^. Also, altered blood concentrations of glycerol, alanine and arginine point to a general metabolic impairment in *Plasmodium*-infected patients^42–44^. Hypoglycemia in patients with malaria can, on the one hand, be explained by an increased glucose uptake by the parasite and the febrile host. On the other hand, host glucose production may be impaired^45^. Here, we clearly demonstrate that in murine malaria models, adrenal hormones promote host survival and prevent the development of severe hypoglycemia upon infection, independently of the parasite burden. The glucose counter-regulatory effects of adrenal hormones are thus essential to survive malaria. Hypoglycemia has been described in other studies of murine malaria models^46–50^ and the group of Elased et al. even ascribed it to increased insulin levels^46,51^. In this study, we show that insulin is not driving the hypoglycemia, since insulin levels were not increased in infected Adx mice and potent inhibition of insulin release by clonidine could not reverse the hypoglycemia. Of note, in malaria patients, hypoglycemia only develops in an insulin-dependent manner upon quinine treatment (quinine induces insulin), whereas in patients not treated with quinine, life-threatening hypoglycemia also develops in an insulin-independent way, similar to our observations in mice^52^.

Adrenalin and GCs both regulate glucose homeostasis during conditions of stress. In rats, adrenalectomy increases the glycolytic flux, decreases gluconeogenesis and rapidly depletes glycogen stores in brain astrocytes^53^. GCs stimulate hepatic glycogen synthesis and gluconeogenesis^54^. In particular, fasting-induced hepatic expression of the gluconeogenic enzymes G6Pase and PEPCK is dependent on GC action, and DEX treatment typically induces the expression of these molecules^55^. PGC-1α is a crucial coactivator of the GC receptor in the activation of the gluconeogenic program in the liver, and can also be induced by glucagon^56^. Since malaria infection in mice results in anorexia-associated weight loss^33,35^, and in view of the increased metabolic demands and elevated corticosterone levels, one would expect increased expression of gluconeogenic enzymes and PGC-1α upon infection of Sham mice. However, this was not the case. Moreover, and in contrast to expectations, the expression of PGC-1α and gluconeogenic enzymes was not lower in Adx versus Sham mice upon infection. This indicates that the mechanism by which adrenal hormones protect against hypoglycemia in malaria is not through induction of the expression of these gluconeogenic molecules. In addition, it is important to note that neither the high glucagon levels nor DEX treatment of infected Adx mice were able to induce the transcription of these genes, suggesting that malaria-infection severely compromises the induction of their expression. Within the context of an apparently deficient gluconeogenic response, hepatic glycogen stores were consumed upon infection and, in the hypoglycemic Adx mice, completely exhausted. Together, the complete glycogen depletion, deficient gluconeogenic program in the liver and increased inflammatory activity, plausibly account for the lethal hypoglycemia in the infected Adx mice.

The cause of lethality is thought to be brain-mediated since neurological signs were observed (Supplementary Movie 1). The observed hypoglycemia is a likely trigger of these signs, since glucose is an obligate fuel for the brain, and neuronal death occurs if severe hypoglycemia reaches levels below 20 mg/dL^57^. The hypoglycemia is paralleled by increased expression of cytokines, chemokines and iNOS in the brain of infected Adx mice. The enhanced expression of chemokines may have contributed to the increased numbers of CD4^+^ T cells and neutrophils. The increase in iNOS was thus not sufficient to counterbalance the loss of tolerance or to prevent infiltration of CD4^+^ T cells, as previously described by Jeney et al^30^. Its increase might thus be a consequence of the increased brain inflammation. The increased pro-inflammatory expression either directly resulted from the lack of anti-inflammatory GC action or might have been indirectly mediated by the hypoglycemia. Microglia respond to various stressors, including both malaria infection and hypoglycemia^58–60^. Upon activation, they release NO, ROS and pro-inflammatory cytokines such as IL-1 and TNF-α^61^. This microglial activation is reported to be GC-sensitive^62,63^. Moreover, the cerebral production of cytokines may increase the energy demands of the brain, potentially further depleting circulating glucose levels. The inability of TNF-α neutralization to restore normoglycemia or survival may suggest that TNF-α-mediated inflammation is not the main cause of death and may instead be a consequence of the hypoglycemia. Nevertheless, other cytokines may mediate the inflammation, which may also be involved in the increased glucose consumption. This interrelation between inflammation and glycemia is further supported by a decrease in brain inflammation and restoration of normoglycemia with DEX treatment of infected Adx mice, despite the inability of DEX to induce the expression of gluconeogenic genes in the liver.

Various bacterial and viral species stimulate the HPA axis and GC production, similar to infection with *Plasmodium*. Interference with the HPA axis is detrimental during infection with murine cytomegalovirus or with bacteria that produce toxins such as *C. difficile* toxin A, Shiga toxin 2 or superantigen SEB^24^. Similarly, infections or endotoxin may be lethal in patients with adrenal insufficiency or in Adx animals^64^. The mechanism is ascribed to the anti-inflammatory effects of GCs, suppressing an immune system overshoot. It is demonstrated that the protective effect of GCs in septic shock relates to the suppression of IL-1β expression in macrophages and IL-12 production in DCs^65,66^. Our data suggest that GC effects on metabolic processes might have been overlooked as a survival mechanism. Furthermore, our findings might have implications for the mechanisms underlying an Addisonian crisis.

In conclusion, our study identifies a crucial role for adrenal hormones in mediating tolerance to malaria. This effect appears to be mediated by maintenance of normoglycemia and prevention of cerebral and systemic inflammation. Our findings enhance the understanding of metabolic homeostasis in malaria and the consequences of glucose imbalance. This is particularly important, since metabolic disturbances, including hypoglycemia, are common in malaria but the underlying mechanism is insufficiently studied.

## Methods

### Mice

In all experiments, 8 to10 week old male and female mice were used and each experimental and control group contained similar numbers of each sex.

Sham-operated and Adx C57BL/6 J or BALB/c mice were purchased from Janvier (Le Genest-St. Isle, France). Mice were allowed (at least 2 weeks) to recover from the surgery before starting the experiments. Adrenalectomy was confirmed by post-mortem examination and by measurement of plasma corticosterone levels (Supplementary Fig. 1a,b). The drinking water of Adx mice was supplemented with 0.9% NaCl.

All experiments were performed at the KU Leuven according to the regulations of the European Union (directive 2010/63/EU) and the Belgian Royal Decree of 29 May 2013, and were approved by the Animal Ethics Committee of the KU Leuven (License LA1210186, Belgium).

### Infection of mice and clinical scoring

Mice were housed in a conventional or specific pathogen free (SPF) animal house and were IP injected with 10^4^ infected RBCs^67^. Infection was with one of the following parasite strains: *Pc*AS, *Pb*NK65-E (corresponding to the Edinburgh strain^22^) or *Pb*NK65-NY (corresponding to a cloned line (1556Cl1) of the New York strain^22^). *Pc*AS and *Pb*NK65-E were originally kind gifts from the late Prof. Dr. D. Walliker (University of Edinburgh, Scotland, UK)^21^ and *Pb*NK65-NY was a kind gift of Prof. C.J. Janse (Leiden University Medical Center, The Netherlands). Mice received high energy food ad libitum and drinking water was supplemented with para-amino benzoic acid (PABA; Sigma-Aldrich, Bornem, Belgium) to facilitate in vivo parasite growth^21^.

Body weights, parasitemia levels, and clinical scores were determined. The clinical parameters including spontaneous activity (SA), limb grasping (LG), body tone (BT), trunk curl (TC), pilo-erection (PE), shivering (Sh), abnormal breathing (AB), dehydration (D), incontinence (I) and paralysis (P) were evaluated daily to calculate a total clinical score of disease severity. A disease score was given of 0 (absent) or 1 (present) for TC, PE, Sh and AB and 0 (normal), 1 (intermediate) or 2 (most serious) for the other parameters. The total clinical score was calculated by the following formula: SA + LG + BT + TC + PE + 3*(Sh + AB + D + I + P). Peripheral parasitemia (percentage of RBCs that are infected) was determined by microscopic analysis of blood smears after Giemsa staining (VWR, Leuven, Belgium). Blood glucose and lactate levels were measured in tail blood with the use of, respectively, a OneTouch Verio glucometer (LifeScan, Zurich, Switzerland) and a Lactate Plus meter (Nova Biomedical, Waltham, Massachusetts, USA). Mice were euthanized with Dolethal (Vétoquinol, Aartselaar, Belgium; 200 mg/mL, IP injection of 50 µL) when the humane endpoints were reached (clinical score of ≥ 15) or at the indicated time points.

### Treatments

Glucose complementation was by addition of D-glucose (Sigma-Aldrich) in the drinking water of the mice at a concentration of 5% (wt/vol), starting 5 days after infection. The non-treated group received water without glucose. Morning and afternoon blood glucose levels were measured. If the glycemia dropped below 100 mg/dL in the glucose-treated mice, glucose was supplemented by IP injection (2 g/kg D-glucose in water) and 30 min after injection, blood glucose levels were again measured.

Dexamethasone treatment was daily, starting at 4 or 5 days after infection, by IP injection of 200 µl of dexamethasone sodium phosphate (DEX; Sigma-Aldrich), dissolved in phosphate buffered saline (PBS) at a dosage of 3 mg/kg. Controls received PBS by IP injection.

Daily clonidine treatment was started 6 days p.i. by IP injection of a dose of 0.5 mg/kg clonidine hydrochloride (Sigma-Aldrich) dissolved in PBS. In mice with a glycemia below 100 mg/dl, a dosage of 1 mg/kg clonidine was used. Control mice received the corresponding volume of PBS.

To neutralize TNF-α, mice were treated with 0.5 mg rat anti-mouse TNF-α IgG1, clone MP6-XT22 (Biolegend, San Diego, California, USA), dissolved in 500 µL PBS. A morning and evening IP injection of 250 µL were given on day 5, 8 and 11 after infection. Control mice received Rat IgG1 isotype control, clone RTK2071 (Biolegend).

### Collection of tissues

Following sacrifice, blood was collected by heart puncture. The left lung was pinched off and BALF was collected from the right lung^21^. Mice were systemically perfused (transcardial route) with PBS to remove circulating blood. Left lungs, liver, spleen, kidney and brain were removed, weighted and stored at -80 °C until further analysis. Alternatively, brain tissue was fixed in 6% paraformaldehyde for 48 h at 4 °C for histological analysis or further processed for flow cytometry.

### Analyses of biological fluids

Liver function was assessed by measurement of plasma ALT using a reagent set according to the manufacturer’s protocols (Teco Diagnostics, California, USA)^20^.

Urine was collected in a 1.5 mL Eppendorf tube after restraining the mice. Kidney function was assessed by determining the albumin/creatinine ratio in the urine. Albumin was measured by Enzyme Linked Immunosorbent Assay (ELISA) with antibodies and standard from ICL (Immunology Consultants Laboratory, Portland, USA). In all, 96-well NUNC immunoplates were coated overnight at 4 °C with 3 µg/mL of capture antibody in 0.1 M NaHCO_3_, pH 9.6. After washing, the wells were blocked for 2 h at room temperature (RT) with 0.5% casein and 0.1% Tween 20 in PBS. Wells were washed and serial dilutions of urine samples and albumin standard were added to the coated plate and incubated for 2 h at RT. Plates were washed and 1/10,000 horseradish peroxidase (HRP)-conjugated anti-mouse albumin antibody was added to the wells. After incubation for 2 h at RT, the plates were washed. HRP activity was visualized by adding 0.55 µM tetramethylbenzidine (TMB, Sigma-Aldrich) in 0.004% H_2_O_2_ (Merck, Darmstadt, Germany), 0.1 M citrated acetate buffer pH 4.3. The reaction was stopped with 1 M H_2_SO_4_ and optical densities were determined at 450 nm on a microplate spectrophotometer (Power Wave XS, Biotek, Winooski, VT, USA). Creatinine was determined with a Quantichrom Creatinine Assay Kit (DICT-500, Bioassay systems) following the manufacturer’s instructions.

Hemoglobin concentrations were determined by the ‘SDS-haemichrome’ colorimetric assay^68^. Blood was collected from the tail vein into lithium-heparin-coated microvette tubes (Sarstedt, Hoogstraten, Belgium) and diluted in 0.1% SDS, 154 mM NaCl buffer. The absorbance was measured at 536 nm and the concentration was calculated from a standard curve of human hemoglobin (Sigma-Aldrich, St. Louis, MO, USA).

BALF samples were centrifuged and protein concentrations of the supernatants were determined by Bradford assay (Bio-Rad, Hercules, CA, USA).

Blood samples were centrifuged and plasma glucose concentrations were measured using a OneTouch Verio meter. Plasma insulin and glucagon levels were assayed using ELISA (Mercodia, Uppsala, Sweden) according to the manufacturer’s protocols. Plasma corticosterone concentrations were determined using a radioimmunoassay (RIA) with [^3^H]-corticosterone as described previously^69^. Plasma levels of TNF-α, IFN-γ, IL-6, IL-10, CXCL-10, CCL-2 and IL-4 were determined with a ProcartaPlex Multiplex immunassay (Thermo Fisher Scientific Inc., Waltham, MA, USA) according to the manufacturer’s protocol. Plasma FFA concentrations were measured using a colorimetric FFA quantification Assay Kit (Abcam, Cambridge, UK) according to the manufacturer’s instructions.

### Hepatic glycogen measurement

Liver homogenates (10 mg/100 µL) were incubated at 100 °C for 5 min and cleared by centrifugation at 13,000 g for 10 min. A colorimetric Glycogen Assay Kit (Sigma Aldrich) was used according to the manufacturer’s instructions.

### Quantitative reverse transcription-polymerase chain reaction

The left lung, liver lobe and the spleen were mechanically homogenized in RLT buffer from the RNeasy kit. QIAzol lysis reagent (Qiagen, Hilden, Germany) was used to homogenize brain tissue. The brain homogenate was separated into aqueous RNA-containing and organic phases by chloroform addition and centrifugation. RNA was extracted with the RNeasy Mini Kit (Qiagen) according to the manufacturer’s guidelines with quantification by UV absorption. For each sample, cDNA was synthesized using the High Capacity cDNA Reverse Transcription Kit (Applied Biosystems) and quantitative reverse transcription-polymerase chain reaction (qRT-PCR) was performed on 25 ng and 12.5 ng cDNA with primer and probe sets from Integrated DNA Technologies (IDT, Leuven, Belgium) using an ABI Prism 7500 Sequence Detection System (Applied Biosystems). Data were normalized to the non-infected control Sham and 18 S ribosomal RNA levels^70^. All used primers are listed in Supplementary Table 1.

### Flow cytometry

Leukocytes were isolated from brains^71^. After cardial perfusion with 40 mL of ice-cold PBS, brains were collected in Hank’s balanced salt solution (HBSS, Gibco/ Thermo Fisher Scientific Inc.) supplemented with 0.075% (w/v) sodium bicarbonate. The organs were minced with scissors, followed by a 15 min digestion at 37 °C with 2 mg/mL collagenase D (Roche, Manheim, Germany) and 14 µg/mL DNase I (Roche). Thereafter, digested tissues were filtered through a 100 µm cell strainer (VWR, Heverlee, Belgium) and washed with HBSS. The cell pellet was resuspended in 10 mL 37% (v/v) Percoll (GE healthcare, Upsala, Sweden) and centrifuged. The layer of myelin was sucked off and the cells were washed, resuspended in PBS supplemented with 2% FCS and counted in a Bürker chamber with trypan blue exclusion.

Before surface staining, single cells were incubated with Fc-receptor blocking antibodies anti-CD16/anti-CD32 (Miltenyi Biotec, Leiden, The Netherlands) and Zombie Aqua Fixable Viability Dye (BioLegend). Cells were washed and incubated for 25 min with rabbit anti-mouse Tmem119 (106-6; Abcam) in PBS supplemented with 2% FCS. Subsequently, cells were washed three times and stained in Brilliant stain buffer (BD Biosciences Erembodegem, Belgium) for 20 min with the following monoclonal antibodies: anti-CD3 (FITC, 145-2c11, Biolegend), anti-CD4 (APC eFluor 780, RM4-5, eBioscience), anti-CD8a (BV711, 53-6.7, BD Biosciences), anti-CD11b (PerCP-Cy5.5, M1/70, eBioscience), anti-CD45 (BUV395, 30-F11, BD Biosciences), anti-F4/80 (BV785, BM8, Biolegend), anti-Ly6G (PE, 1A8, eBioscience), anti-MHC class II (Pe-Cy7, M5/114.15.2, Biolegend) and secondary donkey anti-rabbit (Alexa Fluor 647, Poly4064, Biolegend). Cells were washed and fixed with 0.4% formaldehyde in PBS. Samples were analysed with a LSR Fortessa X-20 using FACSDiva software (BD Biosciences). Live singlet cells (ZombieAqua-) were analyzed with FlowJo (Version 10). To calculate absolute cell numbers, the total number of counted leukocytes was multiplied with the percentage of CD45^+^ cells for each cell subset.

### Histological analysis

After fixation, tissues were embedded in paraffin. Immunohistochemistry analysis was performed on paraffin-embedded sections with anti-CD45 monoclonal antibodies (Clone 30-F11, BD Biosciences,) using an autostainer (Leica Bond Max, Leica Microsystems, Diegem, Belgium). Paraffin sections were pre-treated with Epitope Retrieval Solution 2 BOND (Leica) for 20 min at 97 °C. The Bond Polymer Refine Detection kit (Leica) containing a peroxide block, 3,3′-diaminobenzidine tetrahydrochloride hydrate (DAB) chromogen and hematoxylin counterstain, was used following the manufacturer’s instructions. Endogenous peroxidase activity was quenched using the provided peroxide block for 5 min. Then, the sections were incubated with the primary antibodies diluted 1/50 in Bond Primary Antibody Diluent (Leica) for 30 min at room temperature. Subsequently, slides were incubated with peroxidase-labelled rabbit anti-rat (Dako, Heverlee, Belgium; dilution 1/75 in 10% normal human serum) for 30 min at room temperature. After reaction with DAB, the complexes were visualized as brown precipitates. Cell nuclei were counterstained with hematoxylin (Bond Polymer Refine Detection kit). Transmitted light images were captured through a 40 × /0.65 Plan-Apochromat objective by a Leica DM 2000 microscope equipped with a DFC 295 camera (Leica), using Leica Application Suite (LAS) software version 4.2. Images (2048 × 1536 pixels) were not further processed.

### Statistical analysis

The GraphPad Prism Software (GraphPad Software, San Diego, CA) was used for all analyses. P-values for the differences between groups were calculated by the non-parametric two-tailed Mann-Whitney U-test. Significance levels for the survival curves were calculated with the Log-rank (Mantel-Cox) Test. Non-parametric two-tailed Spearman correlations were computed. * *p* < 0.05, ** *p* < 0.01, *** *p* < 0.001, ****p < 0.0001. Each set of data originate from at least 2 separate experiments. Each data point shown represents the mean of a duplicate measurement, except for repeated blood glucose measurements during disease course experiments.

## Electronic supplementary material

Supplementary Information

Description of Additional Supplementary Files

Supplementary Movie 1

Reporting Summary

## Data Availability

The authors declare that all data supporting the findings of this study are included in the paper and its supplementary information files. Data available from the author upon reasonable request.
