# Supplementary material for: Adrenal hormones mediate disease tolerance in malaria
Source: Nat Commun. 2018 Oct 30;9:4525. doi: 10.1038/s41467-018-06986-5 (PMC6207723; doi:10.1038/s41467-018-06986-5)
Supplement: Supplementary file 1 — Supplementary Information [file 41467_2018_6986_MOESM1_ESM.pdf]

Supplementary Information for

Adrenal hormones mediate disease tolerance in malaria

Leen Vandermosten[1], Thao-Thy Pham[1], Sofie Knoops[1], Charlotte De Geest[1], Natacha Lays[1], Kristof Van der Molen[1], Christopher J. Kenyon[2], Manu Verma[2], Karen E. Chapman[2], Frans Schuit[3], Karolien De Bosscher[4], Ghislain Opdenakker[1], Philippe E. Van den Steen[1*]

[1] Laboratory of Immunobiology, Department of Microbiology and Immunology, Rega Institute for Medical Research, KU Leuven, Leuven 3000, Belgium

[2] Centre for Cardiovascular Science, The Queen's Medical Research Institute, University of Edinburgh, Edinburgh EH16 4TJ, United Kingdom

[3] Gene Expression Unit, Department of Cellular and Molecular Medicine, KU Leuven, Leuven 3000, Belgium.

[4] Receptor Research Laboratories, Nuclear Receptor Lab, VIB Center for Medical Biotechnology, Ghent University, Gent 9000, Belgium

* Corresponding author. email: philippe.vandensteen@kuleuven.be Address: Laboratory of Immunobiology, Rega Institute for Medical Research, KU Leuven, Herestraat 49 box 1044, 3000 Leuven, Belgium

This file includes:

Supplementary Figures 1 to 8

Supplementary Table 1

SUPPLEMENTARY FIGURES

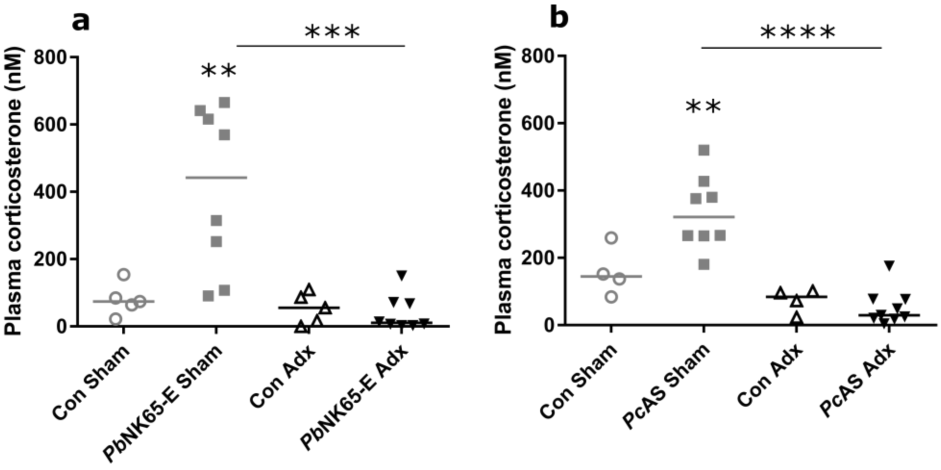

Supplementary Figure 1 | Corticosterone levels are not increased in infected Adx mice.

Sham and Adx BALB/c mice or C57BL/6 mice were infected with *Pb*NK65-E (a) or *Pc*AS (b), respectively. Infected mice were euthanized and dissected at 6 to 7 days p.i. (*Pb*NK65-E) or 8 to 10 days p.i. (*Pc*AS). Plasma corticosterone levels were determined by radioimmunoassay. Each symbol represents data from an individual mouse. Horizontal lines in between data points represent group medians and analysis was by Mann-Whitney U-test. Horizontal lines with asterisks on top indicate statistically significant differences between the indicated groups. Asterisks above individual data sets indicate statistically significant differences compared to the uninfected control group. Data from two separate experiments. ** p<0.01, *** p<0.001, ****p<0.0001

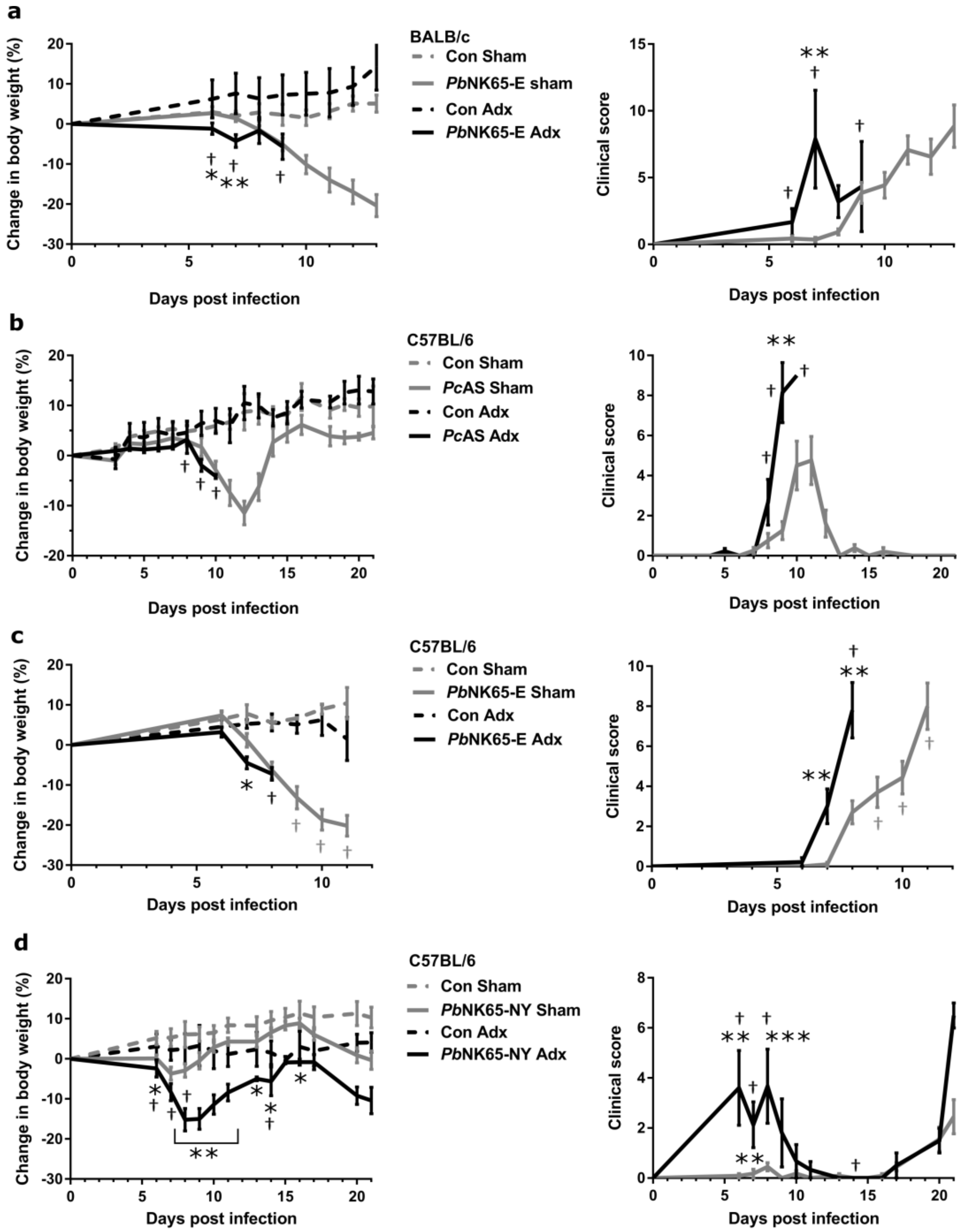

Supplementary Figure 2 | Adrenalectomy results in a higher malaria disease score and body weight loss following infection.

Sham and Adx BALB/c mice (a) or C57BL/6 mice (b-d) were infected with *Pb*NK65-E (a and c), *Pc*AS (b) or *Pb*NK65-NY (d). The relative changes in body weight, compared to day 0, were determined at the indicated time points. A clinical score of the disease severity was calculated based on several clinical parameters as described in the methods. Data are means ± SEM and analysis was by Mann-Whitney U-test. Graphs represent 2 or 3 separate experiments and the numbers of mice are as follows: (a) Sham, n = 14; Adx, n = 13; (b) Sham, n = 8; Adx, n = 9; (c) Sham, n = 10; Adx, n = 9; (d) Sham, n = 12; Adx, n = 10. No data are shown of Adx mice where 2 or fewer mice remained alive: after day 9 (a), day 10 (b) or day 8 (c). Asterisks on top indicate a statistically significant difference between Sham and Adx. Daggers (†) indicate when at least one mouse died or was euthanized for ethical reasons. * p<0.05, ** p<0.01, *** p<0.001.

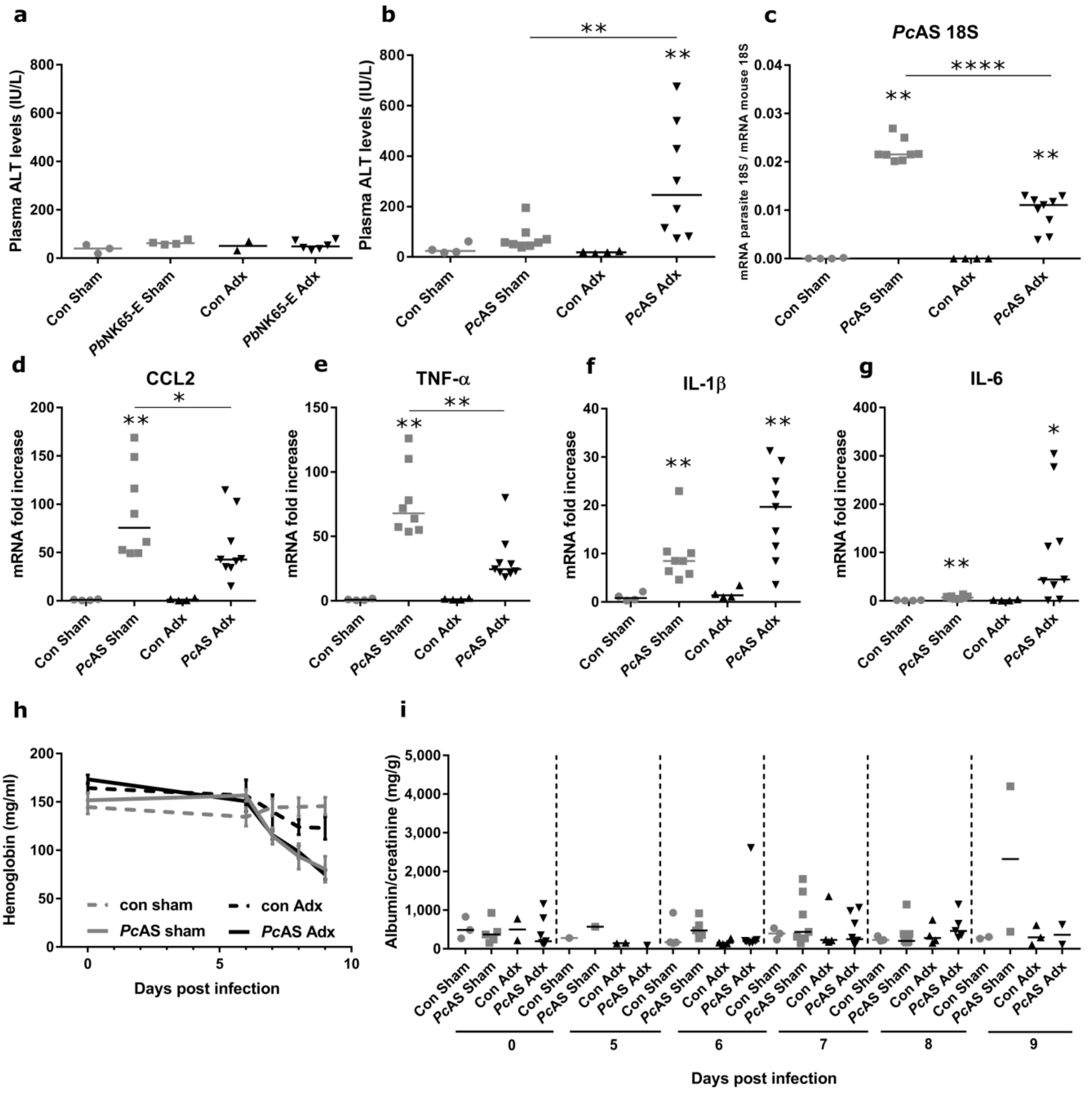

Supplementary Figure 3 | The effect of adrenalectomy on anemia and liver and kidney functions.

Sham and Adx BALB/c mice (a) or C57BL/6 mice (b-i) were infected with *Pb*NK65-E or *Pc*AS, respectively. Infected mice were euthanized and dissected at 6 to 7 days p.i. (*Pb*NK65-E) or 8 to 10 days p.i. (*Pc*AS). (a,b) Alanine aminotransferase (ALT) enzyme levels were measured in the plasma. (c-g) Livers were homogenized and specific mRNA levels were measured by qRT-PCR. Each symbol represents data from an individual mouse. Horizontal lines in between data points represent group medians and analysis was by Mann-Whitney U-test. Horizontal lines with asterisks on top indicate statistically significant differences between the indicated groups. Asterisks above individual data sets indicate statistically significant differences compared to the uninfected control group. (h) Hemoglobin levels were measured in the blood. Data are means ± SEM. Data from two separate experiments. The numbers of mice are as follows: n=4 for controls, n=7-8 for infected. (i) Urinary albumin/creatinine ratio. * p<0.05, ** p<0.01, ****p<0.0001

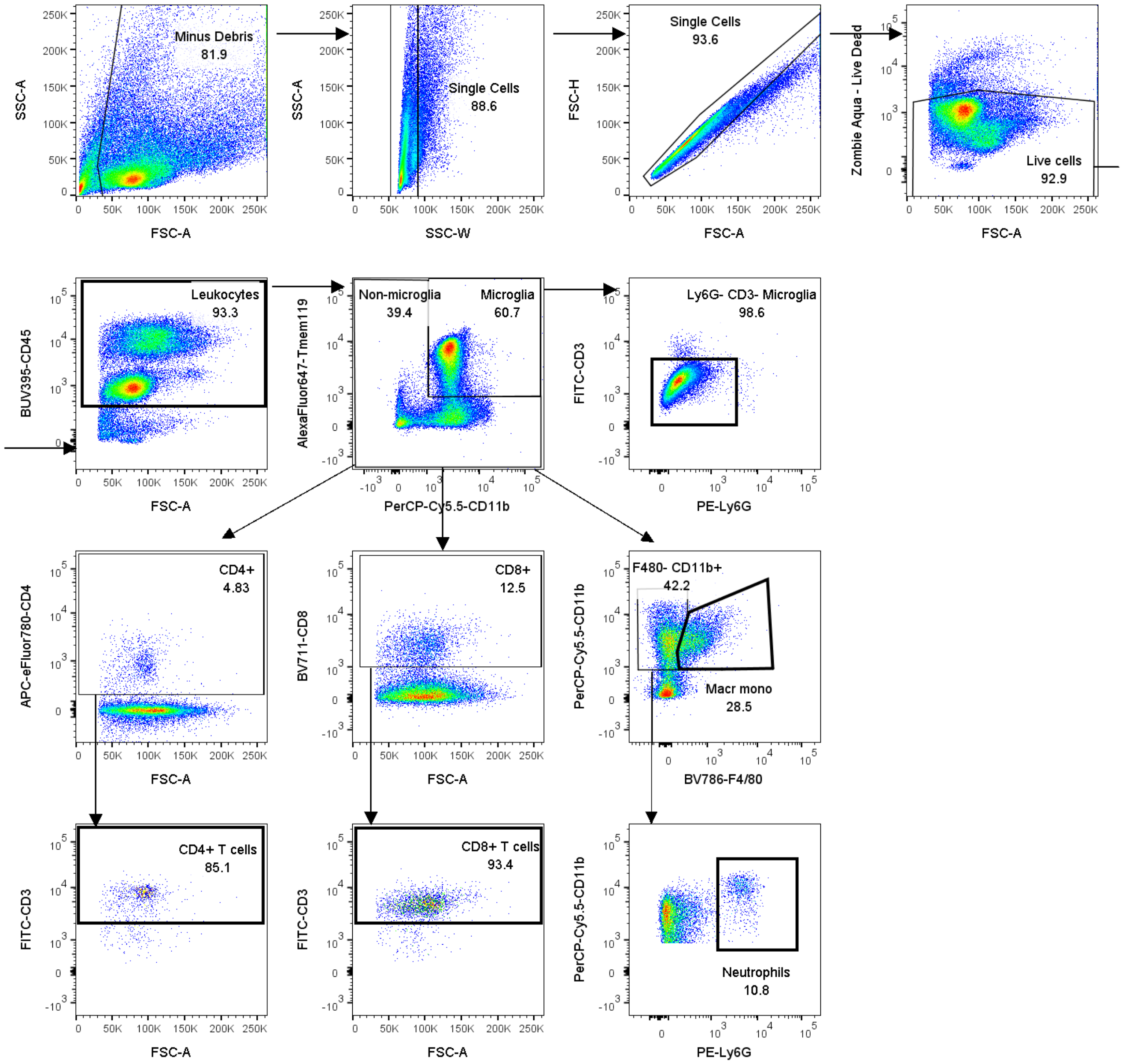

Supplementary Figure 4 | Sequential gating strategy to identify leukocytes in mouse brain.

Leukocytes were isolated from individual brains and analyzed by flow cytometry. After exclusion of debris and doublets, live cells were identified as ZombieAqua negative. Leukocytes were identified by CD45 staining. Microglia were identified as Tmem119$^+$ CD11b$^+$ CD3$^-$ Ly6G$^-$. Non-microglia were differentiated into CD4$^+$ T cells (CD4$^+$ CD3$^+$), CD8$^+$ T cells (CD8$^+$ CD3$^+$), macrophages/monocytes (CD11b$^+$ F4/80$^+$) and neutrophils (CD11b$^+$ Ly6G$^+$). Thick boxes indicate the final gates of the cell populations that are quantified in Figure 4.

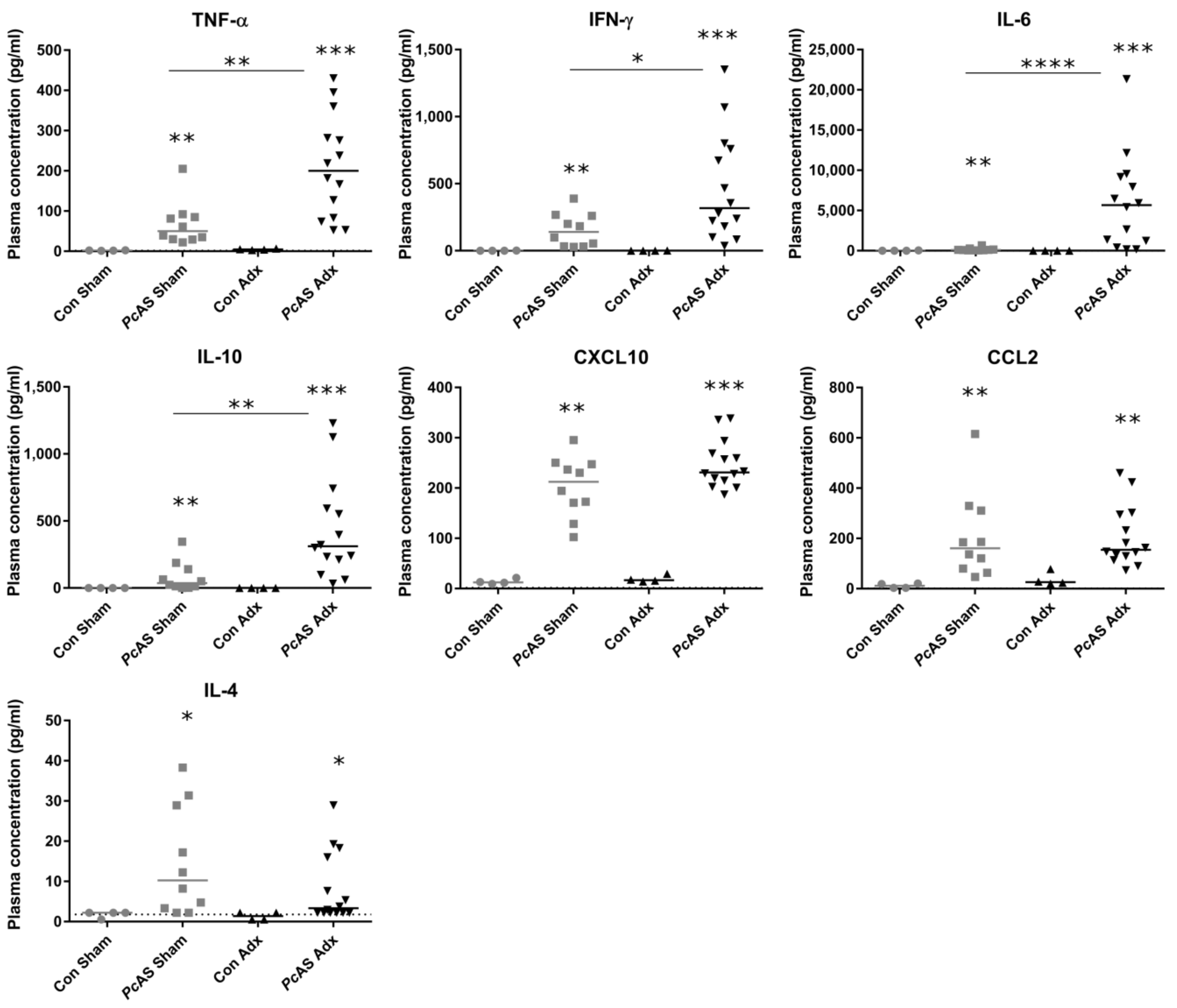

Supplementary Figure 5 | Adrenalectomy results in higher plasma cytokine levels upon infection.

Sham and Adx C57BL/6 mice were infected with *Pc*AS. The mice were euthanized and dissected at 8 to 10 days after infection. Plasma levels of TNF-α, IFN-γ, IL-6, IL-10, CXCL-10, CCL-2 and IL-4 were measured. The dotted line indicates the limit of detection and samples with a measurement below the detection limit were given an arbitrary value of half of the detection limit. Each symbol represents data from an individual mouse. Horizontal lines in between data points represent group medians and analysis was by Mann-Whitney U-test. Horizontal lines with asterisks on top indicate the levels of statistical significance between the indicated groups. Asterisks above individual data sets indicate the levels of statistical significance compared to the uninfected control group. Data from at least two separate experiments. * p<0.05, ** p<0.01, *** p<0.001, ****p<0.0001.

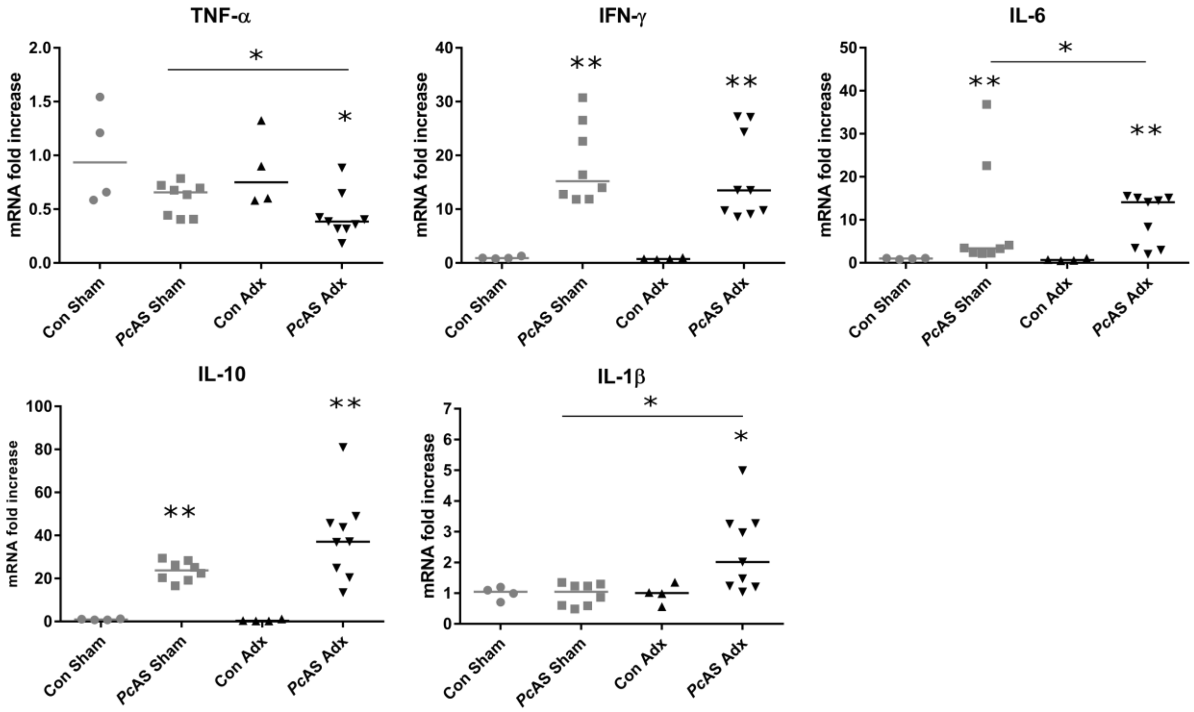

Supplementary Figure 6 | The effect of adrenalectomy on splenic cytokine expression following *Pc*AS-infection of C57BL/6 mice.

Sham and Adx C57BL/6 mice were infected with *Pc*AS. The mice were euthanized and dissected at 8 to 9 days p.i. after which spleens were homogenized and the mRNA levels were analysed by qRT-PCR. Each symbol represents data from an individual mouse. Horizontal lines in between data points represent group medians and analysis was by Mann-Whitney U-test. Horizontal lines with asterisks on top indicate statistically significant differences between infected Sham and Adx mice. Asterisks above individual data sets indicate statistical differences compared to the uninfected control group. Data from two separate experiments. * $p<0.05$, ** $p<0.01$.

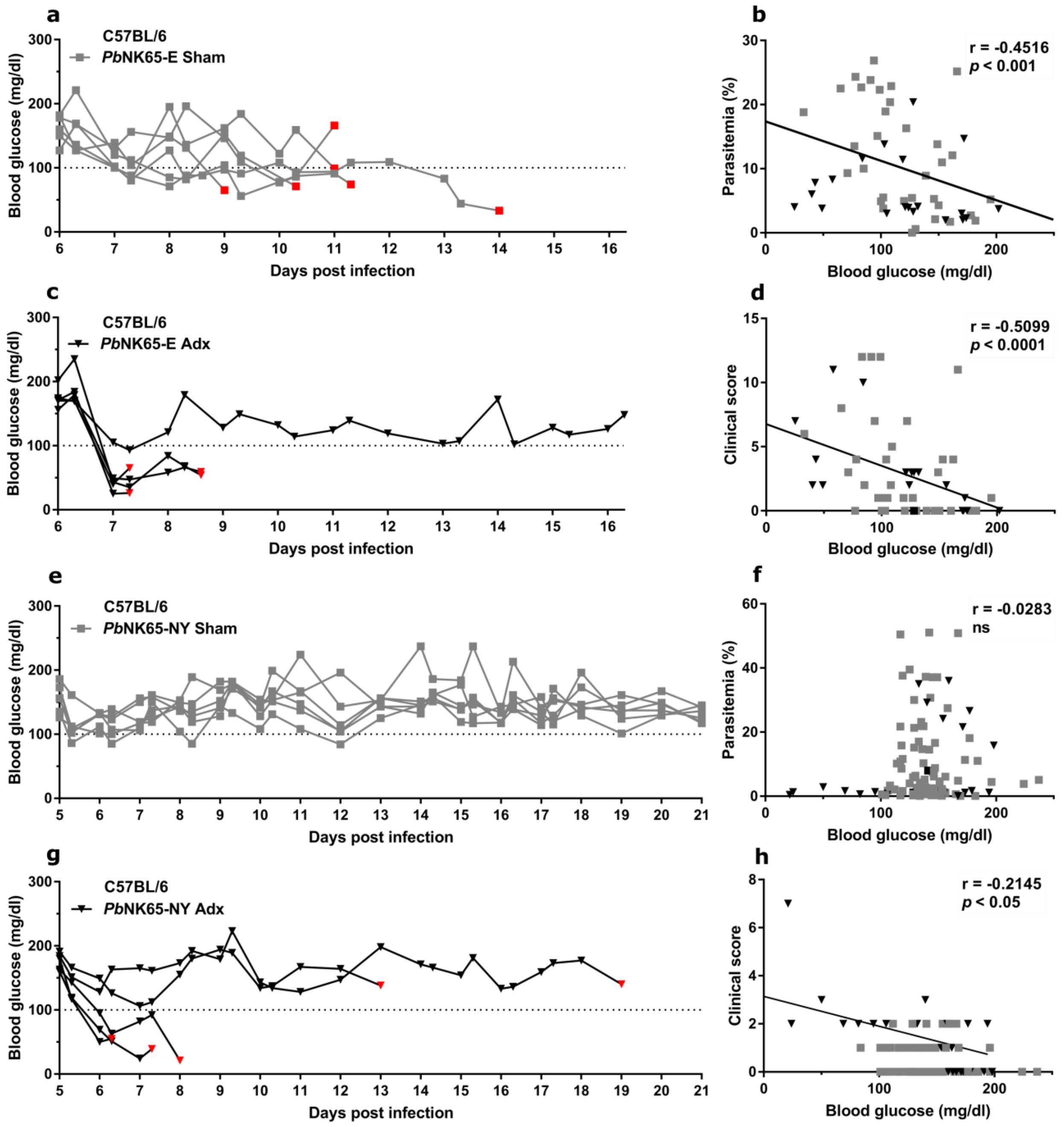

Supplementary Figure 7 | *Pb*NK65-E- and *Pb*NK65-NY-infected Adx C57BL/6 mice develop hypoglycemia early during infection.

Sham and Adx C57BL/6 mice were infected with *Pb*NK65-E (a-d) or *Pb*NK65-NY (e-h). (a,c,e,g) Morning and afternoon blood glucose levels were measured. Each curve represents the glycemia course of an individual mouse. Red dots indicate the last glucose measurement before death. Note that *Pb*NK65-E-infected C57BL/6 mice die early from MA-ARDS around 10 days p.i. (b,d,f,h) Spearman correlations between blood glucose levels and parasitemia or clinical score. Spearman r- and *p*-values are shown. The numbers of infected mice are as follows: (a-d) n=6 for Sham, n=5 for Adx; (e-h) n=6 for Sham, n=5 for Adx. Data from one experiment. ns, not significant.

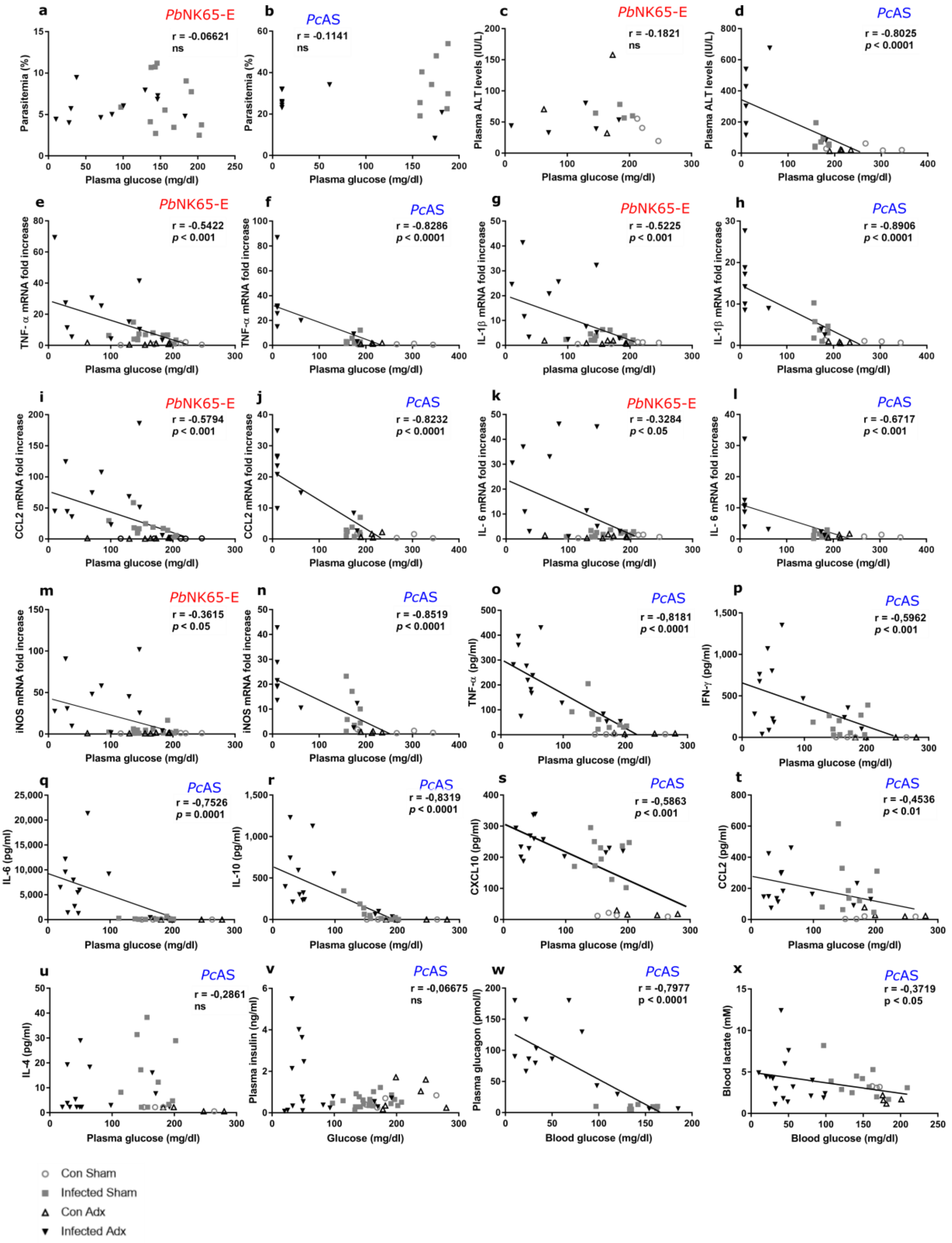

Supplementary Figure 8 | Correlations between glycemia and parasitemia, ALT, cytokines, hormones and lactate.

Sham and Adx BALB/c mice or C57BL/6 mice were infected with *Pb*NK65-E or *Pc*AS, respectively. Infected mice were euthanized and dissected at 6 to 7 days p.i. (*Pb*NK65-E) or 8 to 10 days p.i. (*Pc*AS). Spearman correlations between glucose levels and parasitemia, plasma ALT levels, brain TNF-α, IL-1β, CCL2, IL-6 and iNOS mRNA expression and plasma levels of cytokines, chemokines, insulin and glucagon and blood lactate. Spearman r- and p-values are shown. Data from at least 2 separate experiments. ns, not significant.

SUPPLEMENTARY TABLES

Supplementary Table 1 | List of all qRT-PCR primers

Predesigned qPCR assays (IDT)			
**Name**	**Ref Seq**	**Exon location**	**Assay ID**
CCL2	NM_011333(1)	Exon 1-3	Mm.PT.58.42151692
CXCL10	NM_021274(1)	Exon 1-2	Mm.PT.58.43575827
CXCL6	NM_009141(1)	Exon 1-2	Mm.PT.58.29518961.g
IFN-γ	NM_008337(1)	Exon 1-2	Mm.PT.58.41769240
IL-1β	NM_008361(1)	Exon 1-3	Mm.PT.58.42940223
IL-6	NM_031168(1)	Exon 4-5	Mm.PT.58.10005566
G6Pase	NM_008061(1)	Exon 4-5	Mm.PT.58.11964858
iNOS	NM_010927(1)	Exon 1-2	Mm.PT.58.43705194
NOX2	NM_007807(1)	Exon 8-9	Mm.PT.58.13544805
PEPCK	NM_011044(1)	Exon 3-4	Mm.PT.58.11992693
PGC1α	NM_008904(2)	Exon 5-7b	Mm.PT.58.17390716
TNF-α	NM_013693(1)	Exon 2-4	Mm.PT.58.12575861

Customized primers			
**Name**	**Forward primer**	**Reverse primer**	**6-FAM/ZEN/IBFQ Probe**
*Pb*NK65 18S	5’-CGATAACGAACGAGATCTTAACCT-3’	5’-CGTCAAAACCAATCTCCCAATAAAGG-3’	5’-ACTCGCCGCTAATTAG-3’
*Pc*AS 18S	5’-TAACATGGCTTTGACGGGTAA-3’	5’-TGCTGCCTTCCTTAGATGTG-3’	5’-TCCGGAGAGGGAGCCTGAGAAATA-3’
