# Supplementary material for: Adrenal hormones mediate disease tolerance in malaria
Source: Nat Commun. 2018 Oct 30;9:4525. doi: 10.1038/s41467-018-06986-5 (PMC6207723; doi:10.1038/s41467-018-06986-5)
Supplement: Supplementary file 2 — Description of Additional Supplementary Files [file 41467_2018_6986_MOESM2_ESM.pdf]

Description of Additional Supplementary Files

Supplementary Movie 1

Description: Adrenalectomy induces clinical symptoms after infection with *Pb*NK65-NY Adx (left cage) and sham mice (right cage) were infected with *Pb*NK65-NY. The video was made at 6 days p.i., showing symptoms that indicate brain involvement in Adx mice. Notice that two Adx mice are lethargic and that the third shows a transient stretching phenotype of the hind limbs. This phenotype was consistently observed in infected Adx mice in several experiments. The two lethargic mice reached human endpoints and were euthanized immediately after taking this 2 minute movie.
