# Supplementary material for: Adrenal hormones mediate disease tolerance in malaria
Source: Nat Commun. 2018 Oct 30;9:4525. doi: 10.1038/s41467-018-06986-5 (PMC6207723; doi:10.1038/s41467-018-06986-5)
Supplement: Supplementary file 4 — Reporting Summary [file 41467_2018_6986_MOESM4_ESM.pdf]

nature research

Corresponding author(s): Philippe Van den Steen

Reporting Summary

Nature Research wishes to improve the reproducibility of the work that we publish. This form provides structure for consistency and transparency in reporting. For further information on Nature Research policies, see Authors & Referees and the Editorial Policy Checklist.

Statistical parameters

When statistical analyses are reported, confirm that the following items are present in the relevant location (e.g. figure legend, table legend, main text, or Methods section).

n/a	Confirmed	
☐	☒	The exact sample size (n) for each experimental group/condition, given as a discrete number and unit of measurement
☐	☒	An indication of whether measurements were taken from distinct samples or whether the same sample was measured repeatedly
☐	☒	The statistical test(s) used AND whether they are one- or two-sided<br>*Only common tests should be described solely by name; describe more complex techniques in the Methods section.*
☒	☐	A description of all covariates tested
☒	☐	A description of any assumptions or corrections, such as tests of normality and adjustment for multiple comparisons
☐	☒	A full description of the statistics including central tendency (e.g. means) or other basic estimates (e.g. regression coefficient) AND variation (e.g. standard deviation) or associated estimates of uncertainty (e.g. confidence intervals)
☐	☒	For null hypothesis testing, the test statistic (e.g. F, t, r) with confidence intervals, effect sizes, degrees of freedom and P value noted<br>*Give P values as exact values whenever suitable.*
☒	☐	For Bayesian analysis, information on the choice of priors and Markov chain Monte Carlo settings
☒	☐	For hierarchical and complex designs, identification of the appropriate level for tests and full reporting of outcomes
☐	☒	Estimates of effect sizes (e.g. Cohen's d, Pearson's r), indicating how they were calculated
☐	☒	Clearly defined error bars<br>*State explicitly what error bars represent (e.g. SD, SE, CI)*

Our web collection on statistics for biologists may be useful.

Software and code

Policy information about availability of computer code

Data collection	No software was used
Data analysis	GraphPad Prism version 7.04

For manuscripts utilizing custom algorithms or software that are central to the research but not yet described in published literature, software must be made available to editors/reviewers upon request. We strongly encourage code deposition in a community repository (e.g. GitHub). See the Nature Research guidelines for submitting code & software for further information.

Data

Policy information about availability of data

All manuscripts must include a data availability statement. This statement should provide the following information, where applicable:

- Accession codes, unique identifiers, or web links for publicly available datasets
- A list of figures that have associated raw data
- A description of any restrictions on data availability

The authors declare that all data supporting the findings of this study are included in the paper (and its supplementary information files).

April 2018

Field-specific reporting

Please select the best fit for your research. If you are not sure, read the appropriate sections before making your selection.

☒ Life sciences ☐ Behavioural & social sciences ☐ Ecological, evolutionary & environmental sciences

For a reference copy of the document with all sections, see nature.com/authors/policies/ReportingSummary-flat.pdf

Life sciences study design

All studies must disclose on these points even when the disclosure is negative.

Sample size	No sample size calculation was performed. However, based on extensive experience with previous studies, a sample size of around 10 was foreseen. In view of the observed variability and high replicability, this proved to be sufficient.
Data exclusions	Sporadic mice that did not develop parasitemia after infection were excluded from analyses.
Replication	Each experiment was performed at least twice.
Randomization	Mice were randomly allocated into experimental groups with every group containing similar numbers of each sex.
Blinding	Blinding was not relevant for most analyses, since analytical methods were applied on numerically labeled samples. For the interpretation of the histology, blinding was performed.

Reporting for specific materials, systems and methods

Materials & experimental systems

n/a	Involved in the study	
☒	☐	Unique biological materials
☐	☒	Antibodies
☒	☐	Eukaryotic cell lines
☒	☐	Palaeontology
☐	☒	Animals and other organisms
☒	☐	Human research participants

Methods

n/a	Involved in the study	
☒	☐	ChIP-seq
☐	☒	Flow cytometry
☒	☐	MRI-based neuroimaging

Antibodies

Antibodies used

TNF-α neutralizing antibody: Ultra-LEAF™ Purified anti-mouse TNF-α, Biolegend, #506348, MP6-XT22, lot B245130
Isotype control: Ultra-LEAF™ Purified Rat IgG1, κ isotype Ctrl, Biolegend, #400458, RTK2071, lot B252471
Histology: Rat Anti-Mouse CD45, BD Biosciences, #550539, 30-F11, lot 5236810
Albumin ELISA: HRP conjugated goat anti-mouse albumin, ICL, #GAL-90P, Lot 8; Affinity purified goat anti-mouse albumin, ICL, #GAL-90A, Lot 7
Flow cytometry:
anti-CD16, Miltenyi Biotec, #130-092-575, lot 5180111377;
anti-Tmem119, Abcam, #ab210405, 106-6, lot GR304441-9;
FITC anti-CD3, Biolegend, #11-0031-81, 145-2c11, lot 4323281;
APC e Fluor 780 anti-CD4, eBioscience, #47-0042-80, RM4-5, lot 1928691;
BV711 anti-CD8a, BD Biosciences, #563046, 53-6.7, lot 7339855;
PerCP-Cy5.5 anti-CD11b, eBioscience, #45-0112-80, M1/70, lot 4288959;
BUV395 anti-CD45, BD Biosciences, #564279, 30-F11, lot 7177743;
BV785 anti-F4/80, Biolegend, #123141, BM8, lot B248322;
PE anti-Ly6G, eBioscience, #12-9668-82, 1A8, lot 4316530;
Pe-Cy7 anti-MHC class II, Biolegend, #107629, M5/114.15.2, lot B246676;
Alexa Fluor 647 donkey anti-rabbit, Biolegend, #406414, Poly4064, lot B242361

Validation

The MP6-XT22 clone of anti-mouse TNF-α has been validated previously (Plessner et al., 2007 J Infect Dis 195(11): 1643-1650)
The histological staining with anti-mouse CD45 was validated in our lab on sections of the spleen, analogue to the manufacturer's data sheet.
Concentrations of the anti-albumin antibodies were according to the specifications on the manufacturer's product sheets.
Flow cytometry antibodies were titrated on murine leukocytes to define the right concentration. Fluorescence minus one

samples were included for a correct gating. Most markers are well accepted to define the cell populations. The manufacturer's websites provide validation statements for these antibodies. The anti-Tmem119 antibody has been validated in the following study: Bennett et al., 2016 Proc. Natl. Acad. Sci. 113(12): E1738–E1746.

Animals and other organisms

Policy information about studies involving animals; ARRIVE guidelines recommended for reporting animal research

Laboratory animals	Species:<br>- mice, C57BL/6J, male and female, 8-10 weeks<br>- mice, BALB/c, male and female, 8-10 weeks
Wild animals	the study did not involve wild animals
Field-collected samples	the study did not involve samples collected from the field

Flow Cytometry

Plots

Confirm that:

☒ The axis labels state the marker and fluorochrome used (e.g. CD4-FITC).

☒ The axis scales are clearly visible. Include numbers along axes only for bottom left plot of group (a 'group' is an analysis of identical markers).

☒ All plots are contour plots with outliers or pseudocolor plots.

☒ A numerical value for number of cells or percentage (with statistics) is provided.

Methodology

Sample preparation	Leukocytes were isolated from brains. After perfusion with 40 ml of ice-cold PBS, brains were collected in Hank's balanced salt solution (HBSS, Gibco/ Thermo Fisher Scientific Inc.) supplemented with 0.075% (w/v) sodium bicarbonate. The organs were minced with scissors, followed by a 15 min digestion at 37°C with 2 mg/ml collagenase D (Roche, Manheim, Germany) and 14 µg/ml DNase I (Roche). Thereafter, digested tissues were filtered through a 100 µm cell strainer (VWR, Heverlee, Belgium) and washed with HBSS. The cell pellet was resuspended in 10 ml 37% (v/v) Percoll (GE healthcare, Upsala, Sweden) and centrifuged. The layer of myelin was sucked off and the cells were washed, resuspended in PBS supplemented with 2% FCS and counted in a Bürker chamber with trypan blue exclusion.<br>Before surface staining, single cells were incubated with Fc-receptor blocking antibodies anti-CD16/anti-CD32 (Miltenyi Biotec, Leiden, The Netherlands) and Zombie Aqua Fixable Viability Dye (BioLegend). Cells were washed and incubated for 25 min with rabbit anti-mouse Tmem119 (106-6; Abcam) in PBS supplemented with 2% FCS. Subsequently, cells were washed three times and stained in Brilliant stain buffer (BD Biosciences Erembodegem, Belgium) for 20 min with the following monoclonal antibodies: anti-CD3 (FITC, 145-2c11, Biolegend), anti-CD4 (APC eFluor 780, RM4-5, eBioscience), anti-CD8a (BV711, 53-6.7, BD Biosciences), anti-CD11b (PerCP-Cy5.5, M1/70, eBioscience), anti-CD45 (BUV395, 30-F11, BD Biosciences), anti-F4/80 (BV785, BM8, Biolegend), anti-Ly6G (PE, 1A8, eBioscience), anti-MHC class II (Pe-Cy7, M5/114.15.2, Biolegend) and secondary donkey anti-rabbit (Alexa Fluor 647, Poly4064, Biolegend). Cells were washed and fixed with 0.4% formaldehyde in PBS.
Instrument	LSR Fortessa X-20 using FACSDiva software (BD Biosciences)
Software	FlowJo (Version 10)
Cell population abundance	No sorting was performed.
Gating strategy	Leukocytes were isolated from individual brains and analyzed by flow cytometry. After exclusion of doublets and debris by FSC and SSC, live cells were identified as ZombieAqua negative. Leukocytes were identified by CD45 staining. Microglia were identified as Tmem119+ and CD11b+ and were further confirmed to be CD3- and Ly6G-. Non-microglia were differentiated into CD4+ T cells (CD4+ CD3+), CD8+ T cells (CD8+ CD3+), Macr/mono (CD11b+ F4/80+) and neutrophils (CD11b+ Ly6G+). The boundaries between positive and negative staining were defined based on fluorescence minus one samples.

☒ Tick this box to confirm that a figure exemplifying the gating strategy is provided in the Supplementary Information.
